# Strategies for remediating the impact of math anxiety on high school math performance

**DOI:** 10.1038/s41539-023-00188-5

**Published:** 2023-10-02

**Authors:** Rachel G. Pizzie, David J. M. Kraemer

**Affiliations:** 1 Gallaudet University, Washington, DC USA; 2 Dartmouth College, Hanover, NH USA

**Keywords:** Human behaviour, Education

## Abstract

Students with math anxiety experience excessive levels of negative emotion, including intrusive and distracting thoughts, when attempting to learn about math or complete a math assignment. Consequently, math anxiety is associated with maladaptive study skills, such as avoidance of homework and test preparation, creating significant impediments for students to fulfill their potential in math classes. To combat the impact of math anxiety on academic performance, we introduced two classroom-based interventions across two samples of high school math students: one intervention focused on emotion regulation (ER) using cognitive reappraisal, a technique for reframing an anxious situation, and the other intervention encouraged students to improve their study habits. The Study Skills (SS) intervention was associated with increased grades for highly anxious students during the intervention period, whereas the ER intervention was less efficacious in countering anxiety-related decreases in grade performance. The SS intervention encouraged highly math-anxious students to incorporate self-testing and overcome avoidant behaviors, increasing academic performance and ameliorating performance deficits associated with increased anxiety that were observed in both groups prior to intervention, and that persisted in the ER group. Notably, the benefits observed for the SS group extended to the post-intervention quarter, indicating the potential lasting effects of this intervention. These results support the hypothesis that using better study strategies and encouraging more frequent engagement with math resources would help highly-anxious students habituate to their math anxiety and ameliorate the negative effects of anxiety on performance, ultimately increasing their math comprehension and academic achievement.

Anxiety in the classroom can have significant negative effects on students’ well-being and academic performance^1,2^. Math anxiety is hypothesized to impact academic performance through two mechanisms: students’ performance is impacted by distraction from the task at hand through pervasive, intrusive anxious thoughts, or by a failure to develop effective study techniques such that the information cannot be easily accessed during a high-pressure testing scenario^1,2^. In the present study, we compare two classroom interventions targeted at ameliorating the deficits associated with math anxiety in high school math classrooms: one intervention focused on emotion regulation techniques^3^ targeting the negative thoughts that distract from performance, and another intervention focused on improving study skills through “desirable difficulties” such as self-testing^4^.

Previous research has suggested that math anxiety is negatively associated with math performance through intrusive worries that co-opt working memory resources, detracting cognitive resources from the task at hand^5–7^. Here we propose two different intervention techniques that would target anxiety in different ways. In an Emotion Regulation (ER) intervention strategy, students learn techniques to reframe or rethink their negative and anxious emotional experience while engaging with math. In this way, students learn how to downregulate their anxious response to mathematics, and thereby reduce the negative impact of anxiety on performance^8–10^. In a Study Skills (SS) intervention strategy^11–13^, students learn evidence-based strategies to improve their math studying and learning. This intervention was hypothesized to interact with anxiety in two potential ways: a) by improving learning of mathematical concepts, making them more robust to any anxiety that might detract from performing these skills, and/or b) by helping students to overcome the tendency to avoid mathematics by encouraging students to approach math more often, using better skills like self-testing to learn math, and helping students to habituate their anxiety. Across both the ER and SS interventions, both interventions were designed to ameliorate the negative effects of math anxiety on math performance, and in this study, we compared the effects of both interventions on grades in high school math classes.

The Study Skills intervention was focused on improving study habits through spaced studying and self-testing^14–17^. One potential approach to ameliorating math anxiety is to strengthen students’ study skills and thereby reduce the amount which anxiety interferes in math performance^13,18^. Indeed, maladaptive study behaviors such as avoidance of effortful study strategies contribute to performance deficits in math anxiety^19,20^. Implementing course curricula that emphasize self-testing increases learning, increases test performance, and remediates the negative effects of test anxiety on performance^11,21^. These techniques have all been shown to increase task performance in both laboratory procedures and real-world classroom settings^14,22^, and there is some evidence to suggest that implementing curricula focused on retrieval practice is not only linked to better learning^12,14^ but is also associated with a reduction in anxiety in academic environments^11^. Increased self-testing may serve as a method to reduce negative feelings and reduce the impact of anxiety on performance by providing further instruction in the material, as well as providing increased exposure and desensitization for anxiety. A math-focused study used 8 weeks of individualized tutoring to examine the changes in math anxiety and brain plasticity in children^13^. An additional study that compared improved study skills to an anxiety intervention found that not only did math strategy training result in improved math achievement, but also resulted in a reduction of anxiety^18^. Over the course of this intensive tutoring period, students who showed remediation of math anxiety also showed reduced activity in the amygdala. However, due to the time and potential monetary expense associated with this method, this tutoring intervention would be difficult to implement on a large scale.

In contrast to these promising approaches that are focused on improving student retention of academic content, a separate area of research targeting feelings of anxiety has shown some success in reducing negative affect as well as improving performance^2,9,10,23–25^. Interventions that focus on therapy and alleviating anxiety have been effective in reducing self-reported math anxiety and have varied effectiveness in terms of their effect on mathematical performance. However, individualized therapy can be costly, time-consuming, and outcome measures evaluating math performance have frequently been constrained to tasks completed in the lab. To address these limitations, here we examine the effects of an anxiety-reducing Emotion Regulation strategy in the classroom that is quick, easy, and free to implement.

At the core of many of the therapy-based strategies is an emphasis on emotion regulation^26–28^. Emotion regulation is the mental process designed to change or alter one’s emotional experience, either augmenting a positive response or regulating and reducing negative feelings. Two common emotion regulation techniques include expressive suppression, or hiding one’s feelings^29,30^, and cognitive reappraisal, or rethinking or reframing the context of the emotional experience in order to change or decrease affect^31,32^. Particularly, cognitive reappraisal has been shown to decrease physiological arousal, increase cognitive control and decrease negative emotion. Cognitive reappraisal has been shown to improve reactivity to mathematics for highly math-anxious individuals^9,10,33,34^. In a study exploring the relationship between math accuracy and arousal in high vs. low math anxious individuals, cognitive reappraisal attenuated the relationship between physiological arousal (measured by electrodermal activity, EDA) and math task performance, such that even elevated physiological arousal (likely due to anxiety) was no longer associated with poor performance^10^.

Previous work has utilized neuroimaging to investigate a cognitive reappraisal strategy and its effects on math performance and neural activity in regions of the brain associated with arithmetic processing^9^. Whereas using a cognitive reappraisal strategy was associated with improved performance for highly math-anxious individuals, this was also associated with increased neural activity in regions of the brain associated with arithmetic processing, namely the intraparietal sulcus. These results suggest that not only is cognitive reappraisal associated with increased accuracy for highly math-anxious individuals, but this is also associated with a tandem increase in neural activity in regions of the brain that would support processing mathematical information. Although these results show promising results in a lab setting, the present research was designed to explore whether these cognitive reappraisal strategies would be efficacious in a real-world classroom setting.

Strategies that incorporate aspects of reappraisal have also been effective in improving performance in classrooms, specifically reducing anxiety related to math tests and ameliorating the negative effects of anxiety on performance^33^. Expressive writing reduces the interference created by rumination in math anxiety by allowing students to write about test-related worries before a test or other math task^24,25,35^. This technique provides the ideal context for “cognitive strategies that change the meaning of a stressful situation”^24^, allowing students to rethink or reframe their feelings of anxiety, reducing the negative attributions of the situation.

Following these two separate lines of investigation regarding effective study strategies and emotion regulation techniques, this research is targeted at reducing the deficits in mathematical performance associated with math anxiety by introducing two intervention approaches into real-world high school mathematics classrooms. We sought to test both intervention techniques, one targeting emotion regulation (ER), and the other focusing on utilizing better study skills (SS) in high school mathematics classrooms. One intervention was focused on an approach that should already be somewhat familiar to students – namely, using better study habits when reviewing material and preparing for exams. In fact, there is the possibility that highly math-anxious students will respond negatively to the requirement of increased exposure to math materials that this approach necessitates. The second intervention is aimed directly at reducing the negative feelings that anxious students experience when they encounter math by providing them with techniques to use for regulating their own emotions.

Both intervention techniques were designed to introduce the intervention strategy in small group discussions with high school students at the beginning of the second semester, then students were followed with questionnaires throughout the semester, and both groups completed a short writing task relevant to their assigned technique immediately before the midterm exam^24^. Introduction of these intervention strategies during the second semester allowed us to compare each student to their own pre-intervention class performance. Both intervention strategies were designed to be easy and cost-free to implement in a classroom setting, aiding students by introducing flexible, intuitive strategies that could reduce avoidance of mathematics, and reduce the decline in performance associated with anxiety, thus encouraging students to reach their full potential.

In this research, we aim to address two main questions:

1. *When the intervention is introduced during second semester, do we observe increases in grade performance in either or both intervention groups relative to first semester performance?*
2. *Is one intervention more effective than the other in reducing the negative impact of math anxiety on grade performance?*
  We will also address additional follow-up analyses to address the following questions:
3. *Comparing grades for Quarter 3 and Quarter 4, does the effect of the intervention last across the second semester, even beyond the main active intervention period?*
4. *Do other sources of anxiety, such as trait anxiety or test anxiety, explain our results?*

## Results

### Analysis design

The majority of the analyses in this project were conducted using linear mixed models (LMM), as these models allow us to account for the fixed effects of our experimental effects (i.e., intervention strategy, individual differences in anxiety), and still account for the important random effects inherent to doing research in a real-world educational setting. We evaluated whether course subject (i.e., algebra, geometry), teacher, and school accounted for differences in math grades. Using a LMM evaluating course subject as a fixed factor and random effects accounting for each individual participant, we found significant differences between course subjects on math grades, ***χ***^2^(3) = 37.17, *p* < .001. We used a LMM evaluating teacher as a fixed factor and random effects for each individual participant, we found significant differences between teachers on math grades, ***χ***^2^(6) = 53.87, *p* < .001. We used a LMM evaluating school as a fixed factor and random effects for each individual participant, we did not find significant differences between schools for math grades, ***χ***^2^(1) = 0.30, *p* = 0.58.

As a result, we decided to include course subject and teacher as random effects in our models in order to control for differences in grades created by these factors, and we did not include school as a factor in these models. In addition, we also included previous math grade to control for previous math performance as a within-subject control for math performance before the intervention was implemented. In the following analyses, we evaluated grades during the third and fourth quarter as outcome measures, with random effects accounting for individual differences in participants, course subject, teacher, and previous math class performance. In the Supplementary Material, we included analyses that evaluated quadratic models of math anxiety to account for a potential curvilinear model (negative quadratic) model of anxiety or stress, as has been demonstrated in previous studies^36^. We found that the quadratic models did not account for additional variance above and beyond the linear models included in the main manuscript. In some cases where we directly evaluate scores from before the intervention, or directly use difference scores to calculate the difference from pre-intervention grades to during the intervention, we utilize linear models. For descriptive statistics associated with this dataset, please see Table 1.

**Table 1:** Demographics of combined dataset and each school’s sample.

Measure	Combined Dataset (*N* = 224)			School 1 (*N* = 68)			School 2 (*N* = 156)		
Mean (SD)	All	ER Group	SS Group	All	ER Group	SS Group	All	ER Group	SS Group
*Overall Math Grade*	74.77 (21.73)	75.49 (21.48)	74.30 (22.03)	73.76 (16.42)	71.87 (17.85)	75.76 (14.61)	75.28 (23.97)	77.01 (23.03)	73.71 (24.83)
*Gender N* (% female)	153 (55%)	61 (54%)	62 (55%)	40 (59%)	21 (60%)	19 (58%)	83 (53%)	40 (52%)	43 (54%)
*AAI-Math* (1-5 Scale)	3.02 (.87)	2.99 (.87)	3.04 (.87)	3.32 (.88)	3.19 (.88)	3.46 (.87)	2.86 (.82)	2.88 (.84)	2.85 (.79)
*MARS* (1-5 Scale)	2.49 (.70)	2.52 (.70)	2.46 (.69)	2.56 (.78)	2.45 (.80)	2.68 (.76)	2.45 (.65)	2.56 (.64)	2.35 (.64)
*AAI-Trait* (1-5 Scale)	2.93 (.79)	2.90 (.81)	2.94 (.76)	2.94 (.84)	2.76 (.87)	3.12 (.76)	2.92 (.76)	2.98 (.77)	2.86 (.75)
*STAI-Trait* (1-4 Scale)	2.33 (.52)	2.33 (.52)	2.32 (.52)	2.35 (.56)	2.26 (.56)	2.45 (.56)	2.31 (.50)	2.35 (.50)	2.26 (.50)
*AAI-Test* (1-5 Scale)	3.46 (.73)	3.45 (.73)	3.46 (.73)	3.46 (.78)	3.37 (.78)	3.55 (.78)	3.46 (.70)	3.48 (.69)	3.42 (.70)
*TAI* (1-4 Scale)	2.42 (.74)	2.45 (.72)	2.39 (.77)	2.42 (.72)	2.39 (.72)	2.46 (.73)	2.42 (.76)	2.48 (.72)	2.36 (.78)

### Evaluating the effects of group assignment in pre-intervention semester

**Fig. 1:**
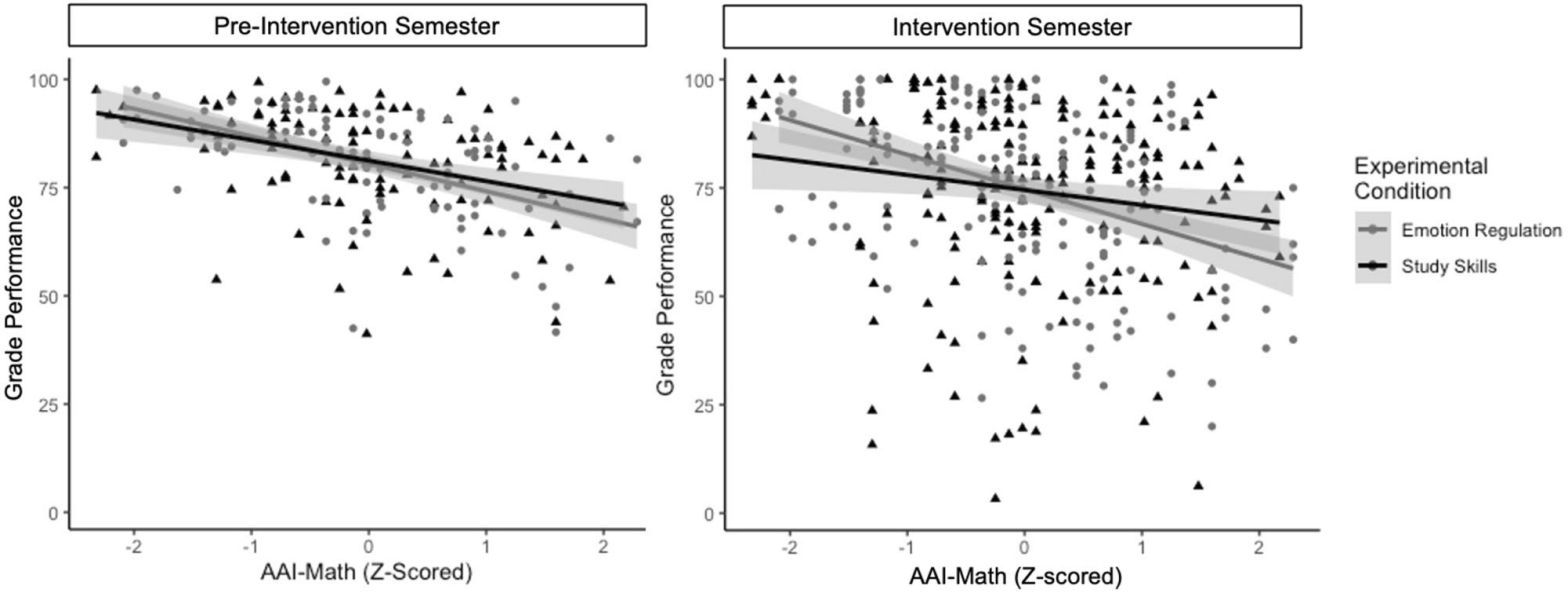
Interaction between math anxiety and intervention group before and during the intervention. Left Before the intervention is introduced, the two groups do not differ on grades, *p* > 0.05, nor is there a significant interaction between group and anxiety on grade performance, *p* > 0.05, however, there is a significant main effect of math anxiety on grades before the intervention was introduced, *F*(1, 192) = 55.69, *p* < 0.001. Right) After the intervention is introduced, we observe a significant interaction between math anxiety and intervention group, collapsing across quarter grades in the second semester (Q3 and Q4 grades), ***χ***^2^(1) = 6.73, *p* = 0.010. For the emotion regulation group, we observe that as MA is increased, grades decrease, indicating that the ER intervention may not have had much effect in reversing the relationship between math anxiety and grade performance. However, for the study skills group, the relationship between math anxiety and grade performance is ameliorated, such that students who have higher anxiety performed better in the study skills group compared to the emotion regulation group. This suggests that the study skills intervention may be more effective at reversing the negative effects of anxiety on performance. Clouds represent standard error bars.

Prior to evaluating the effects of the intervention, we first evaluated whether there were inherent differences between the groups during first semester, *before* the intervention was implemented. Using an average of pre-intervention grades (an average of quarter 1 and quarter 2 grades), we evaluated the differences between intervention groups using a linear model, while accounting for class subject matter and teacher. Before the intervention was implemented, we find a main effect of math anxiety on pre-intervention grades *F*(1192) = 55.69, *p* < 0.001, such that increased math anxiety is associated with decreased grade performance during the pre-intervention semester. When we examine grades before the intervention groups were introduced, there are no significant differences between intervention groups, *F*(1, 192) = 0.16, *p* = 0.69, and there was no interaction between intervention group and math anxiety on pre-intervention grades, *F*(1192) = 1.27, *p* = 0.26. These results confirm that our pseudo-random assignment of students to groups did not inadvertently result in groups that differed substantially in their math performance or effects of math anxiety on performance prior to our study. However, we do observe, as expected, that math anxiety was associated with decreased grade performance across all students before the intervention was introduced (Fig. 1, Left).

### Evaluating effects of the interventions

In developing these interventions, our intention was to create approaches that could be leveraged by students highest in math anxiety, as these are the students who are mostly likely to suffer deficits in math performance. During second semester, we analyzed grades as an outcome measure, we utilized a LMM to evaluate the interaction between math anxiety (AAI-Math) and intervention group as fixed factors, and random effects were included in the model for individual participants, course subject, teacher, and previous math performance. Overall, we observe a significant effect of math anxiety, ***χ***^2^(1) = 8.42, *p* = 0.004, such that increased math anxiety is associated decreased grade performance (***β*** = −3.87, *t*(160.89) = −2.90), as it was during the first semester. While there was no significant main effect of group on grades, ***χ***^2^(1) = 0.003, *p* = 0.95, there was a significant interaction between the AAI-Math scores and intervention group for math grades, ***χ***^2^(1) = 6.73, *p* = 0.010 (Fig. 1, Right). This result indicates that one of the interventions was effective at reducing the negative impact of math anxiety on academic performance, while the other was not.

For the ER intervention group, as AAI-Math scores increase math grade performance is decreased, as was the case prior to the intervention. However, the negative impact of math anxiety on performance is ameliorated in the SS intervention group (***β*** = −3.17, *t*(135.59) = −2.59). For the individuals who were highest in math anxiety, participating in the SS intervention group compared to the ER intervention group was associated with increased math grade performance. On average, for students highest in math anxiety, students in the Study Skills group had grades that were approximately half a letter grade higher compared to their peers that were randomly assigned to the Emotion Regulation group (6.29 grade points, calculated as a difference score between groups in the 4th quartile of anxiety scores). For students lower in math anxiety, these results suggest that the Emotion Regulation Intervention was associated with better math performance – possibly because these students may have already been using their own effective study strategies. Students highest in math anxiety tend to have the lowest grades in these courses, and these results indicate that the students highest in math anxiety are able to improve their grade performance by implementing the approaches provided by the study skills intervention.

To further examine the effect of intervention group, we directly compared grades during the main intervention period (Quarter 3) to previous grade performance during the pre-intervention timeframe by calculating a Pre/Post-Intervention Grade Difference score. In order to get an estimate of pre-intervention grade performance, we calculated an average grade from Quarter 1 and Quarter 2 math grades. Then, we subtracted this value from grades earned during the main intervention quarter (Quarter 3). Overall, all grades decreased over the course of the school year, likely reflecting the increased difficulty of the course content. Therefore, almost all of the observed difference values are negative, because we are subtracting the earlier term grades from the later term grades. Scores around zero or positive scores indicate that the student’s math grade performance was maintained or improved during the intervention quarter compared to the student’s previous performance in the class.

**Fig. 2:**
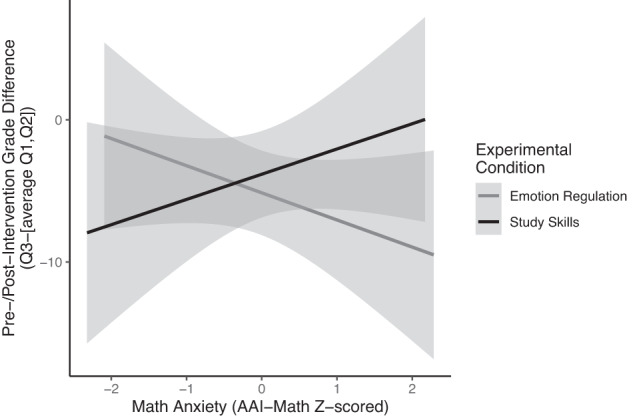
Comparing within-subject changes before and during the intervention. To compare performance during the intervention to students’ own performance before the interventions were introduced, we calculated an Intervention Grade Difference Score. This score is calculated as the difference between students’ math grades during the intervention quarter, subtracting an average of their quarter grades during the previous semester (Q3 – [Average (Q1, Q2)]). We observe an interaction between math anxiety (Z-scored AAI-Math scores) and intervention group on the difference scores, *F*(1,174) = 4.72, *p* = 0.03. For the ER intervention group, we observe a negative relationship between MA and grades, such that more anxious students were more likely to have decreased grades compared to their own previous performance. For the SS intervention group, the pattern was reversed: as math anxiety increased, students were more likely to maintain or improve their grade performance compared to their grade performance before the intervention. Overall, grades in the second semester are lower than those in the first semester, corresponding to the increased difficulty of the math content. Error bars represent standard error.

In this analysis, we used a linear model predicting Pre/Post-Intervention Grade Difference (Q3 – [Average of Q1 and Q2]) as an outcome measure, evaluating the interaction between intervention group and standardized math anxiety score (AAI-Math), and controlling for course subject and teacher (Fig. 2). The intervention groups did not differ in measures of average grade performance (Q1 and Q2 average) before the interventions were introduced, *t*(220.6) = −0.29, *p* = 0.77. Before the intervention groups were introduced, average math grade performance across both groups was equivalent (ER group = 80.88, SS Group = 81.4). There was no significant main effect of math anxiety on Pre-/Post-Intervention Grade Difference scores, *F*(1,174) = 0.01, *p* = 0.93, and no significant main effect of group, *F*(1,174) = 0.39, *p* = 0.53. However, there was a statistically significant interaction between intervention group and math anxiety for the Pre-/Post-Intervention Grade Difference scores, *F*(1,174) = 4.72, *p* = 0.03. For the ER intervention group, higher levels of math anxiety were associated with greater decreases in math grades compared to their previous class performance. Similar to the previous analysis, the negative association observed between Pre-/Post-Intervention Grade Difference scores and anxiety is ameliorated in the SS group (***β*** = −2.19, *t*(174) = −2.17). In the SS intervention group, students who were more highly math-anxious were the most likely to maintain their math grades. Compared to the ER group, the SS group was associated with better grade performance for more highly math-anxious students, directly comparing to students’ own previous grade performance before the introduction of the intervention groups. Using students as their own within-subject controls, we observe that the SS intervention is associated with maintained or better grade performance compared to students enrolled in the ER intervention group for students who experience math anxiety.

### Duration of intervention effects

**Fig. 3:**
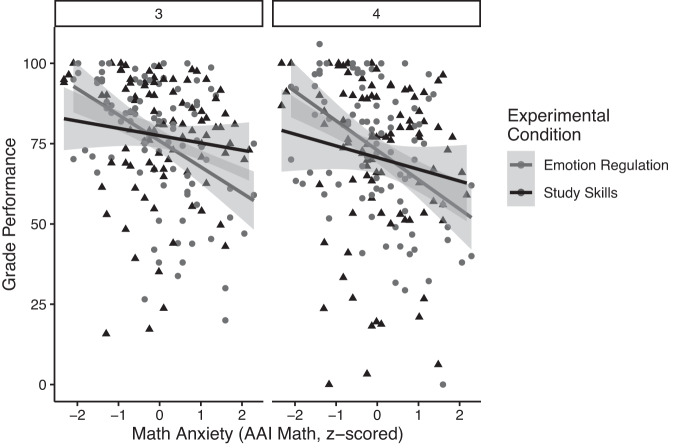
The interaction between group and anxiety is maintained beyond the main intervention quarter across second semester. For second semester grades, following the introduction of the intervention we observe a significant interaction between math anxiety (AAI-Math, z-scored) and intervention group, ***χ***^2^(1) = 6.74, *p* = 0.010. For the Emotion Regulation Group, as math anxiety increases, we observe a characteristic decrease in grade performance. However, for the Study Skills Group, we see that this relationship is ameliorated, such that those higher in math anxiety show improved grade performance compared to those in the ER group. This relationship is maintained across both quarters, such that we do not observe a decrease in the effectiveness of the intervention across time. Between the two quarters of the second semester, the three-way interaction between math anxiety, intervention group, and quarter was not statistically significant, ***χ***^2^(1) = 0.03, *p* = 0.86. Error bars represent standard error.

In order to examine the duration of the effects of the interventions, we also evaluated how math grades were affected by the intervention groups over the full course of second semester by exploring grade performance within each quarter (Fig. 3). We might expect that the interventions would have the greatest effect during the third quarter, while the intervention activity was highest, but then these effects might wane over time, e.g., once the researchers stopped reminding students to utilize their assigned intervention before each exam. Conversely, finding that the effects extended into the fourth quarter would indicate the potential lasting benefits of the interventions. In order to address this question, we conducted a LMM analysis with grades as the outcome measure, exploring the interaction between math anxiety, intervention group, and quarter as fixed factors, and random effects for participants, course subject, teacher, and previous math performance.

In this analysis, we again found a main effect of math anxiety (z-scored AAI-Math scores) on math grades, ***χ***^2^(1) = 8.44, *p* = 0.004, and a main effect of quarter, ***χ***^2^(1) = 24.64, *p* < 0.001, such that overall grades decreased from third to fourth quarter. As before, there was no significant main effect of intervention group on math grades, ***χ***^2^(1) = 0.004, *p* = 0.95. For the post-intervention semester (i.e., 3rd and 4th quarters), there was no significant interaction between math anxiety and quarter, ***χ***^2^(1) = 1.53, *p* = 0.22, such that the detrimental effects of math anxiety were not associated with changes from one quarter to the next. Notably, for math grades, we again observe a significant interaction between math anxiety and intervention group, ***χ***^2^(1) = 6.74, *p* = 0.010, as previously discussed (Fig. 2). There was not a significant interaction between intervention and quarter for math grades, ***χ***^2^(1) = 3.75, *p* = 0.053. Finally, for math grades, there was no significant three-way-interaction between math anxiety, intervention group, and quarter, ***χ***^2^(1) = 0.03, *p* = 0.86. These results confirm that the effects observed in 3rd quarter extend to the 4th quarter. Specifically, the SS intervention was associated with ameliorating math anxiety-related deficits in math class, and this effect was maintained throughout the second semester, including in the quarter after the active intervention period had ended.

### Other sources of anxiety

We also explored whether other sources of anxiety were associated with grade performance or had differential effects based on intervention group. We constructed similar LMMs with grades as the outcome, and utilized fixed factors for intervention group, anxiety, and quarter, with random effects for individual participants, course subject matter, teacher, and previous math performance. In all of these analyses, there were no interactions between anxiety and group, all *p*’s > 0.05. There were no significant two-way interactions between anxiety and quarter, all *p*’s > 0.05, and no significant three-way interactions between other sources of anxiety, intervention group, and quarter, all *p*’s > 0.05. This suggests that the associations between math anxiety and grade performance in these math courses is relatively specific and cannot be accounted for by general experiences of anxiety.

Based on past associations between gender and math anxiety, we also explored whether the effects of math anxiety and group were also associated with gender (coded as a binary: male and female). We constructed a LMM with grades as the outcome, and used fixed factors for intervention group, math anxiety, and gender, with random effects for individual participants, course subject matter, teacher, and previous math performance. There was a main effect of gender on course grades, ***χ***^2^(1) = 10.73, *p* = 0.001, such that female students had higher course grades (*M* = 80.8, *SE* = 5.41, *95% CI*: 65.4–96.2) than male students (*M* = 72.2, *SE* = 5.47, *95% CI*: 57.0–87.5). However, there were no interactions between gender and math anxiety on grades, ***χ***^2^(1) = .23, *p* = 0.63, no interaction between group and gender, ***χ***^2^(1) = 0.01, *p* = 0.89, and no three-way interaction between math anxiety, intervention group, and gender, ***χ***^2^(1) = 0.003, *p* = 0.96.

## Discussion

This study evaluated the relative effects of two interventions on grades in high school math classes, investigating whether either of these interventions might be valuable tools for ameliorating the negative effects of math anxiety on math grade performance. There were no overall differences in grades between the intervention groups, suggesting that for all students, there was not one intervention that resulted in better grade performance than the other. However, improving math class performance for highly math-anxious individuals is a priority. Controlling for previous math performance before the intervention, our results suggest an interaction between math anxiety and Emotion Regulation or Study Skills intervention groups when we evaluate math grades.

Especially for highly math-anxious individuals, individuals assigned to the Study Skills Intervention group had increased math grade performance compared to those assigned to the Emotion Regulation Intervention. For individuals lower in math anxiety, individuals assigned to the Emotion Regulation intervention had better math grades relative to those assigned to the Study Skills intervention. These effects were maintained from the intervention quarter (third quarter) and longitudinally across the rest of the semester (fourth quarter). Compared to previous performance, more highly math-anxious students in the SS intervention were able to maintain or increase their grade performance compared to highly anxious students in the ER intervention, whose grade performance decreased in the intervention quarter. These results suggest that especially for highly math-anxious individuals who are likely to struggle to achieve in math classes, an in-class intervention focused on improving study skills by spaced studying and self-testing resulted in better math grades.

The SS intervention provides a simple, easy-to-administer technique that is intuitive for students to understand, and was effective in increasing math grades. This intervention emphasized self-testing and utilizing practice problems as an effective study strategy, and this technique was associated with better performance for highly math-anxious students, increasing grades up to half a letter grade higher than students assigned to the other intervention. Notably, relative to the ER intervention, the positive effects of the SS intervention were strongest for students who rated at the high end of measures of math anxiety. That these positive effects on grades can be generated from a 20-minute discussion at the beginning of a term, short reminders during study periods and before tests suggests that ameliorating the negative impact of anxiety on mathematics does not necessarily require drastic changes in the classroom.

The results of this intervention are consistent with previous work on utilizing self-testing as a study strategy^11^, and provide further evidence that increases in math grades in study strategies impact those highest in anxiety, increasing their performance^21^. These results also suggest that when students take an active role in implementing changes in their own study techniques – gaining practice in completing math problems and potentially habituating to the effects of anxiety – we see a resulting increase in math grades and a reduction in the effects of anxiety on performance. The impact of the SS intervention had promising results during the intervention quarter, and these effects extend to the subsequent final quarter when reminders about the technique are given, suggesting that students may require continued support, reminders, and implementation of the writing task before testing in order for the positive effects of the intervention to continue.

These results are consistent with our hypotheses that the Study Skills Intervention strategy would help more highly math-anxious students to habituate some of their anxiety, perhaps decreasing the negative impact of math anxiety and improving math performance. Because avoidance is a common feature of math anxiety^19,20^, engaging in better study skills may have helped more highly math-anxious individuals to approach math more often. During the intervention discussions, students often reported difficulty in knowing how to study for their math classes, often rereading the chapter or reviewing notes from class, strategies that have been suggested to be less effective for learning^11^. In this intervention, more highly math-anxious students who may have avoided studying or engaged in maladaptive strategies in the past were encouraged to engage in learning techniques that may have resulted in better math learning, such as spaced studying and self-testing. Our results suggest that encouraging math-anxious students to engage with math more often and use techniques that encourage better learning did not result in exacerbated effects of anxiety. Instead, the Study Skills Intervention was associated with better math grades for more highly math-anxious individuals, providing a boost in math performance to students who would otherwise struggle to achieve in math classes.

Although the ER intervention had limited impact on students, emotion regulation behaviors still seem to play a role in math classes. Although unexpected based on our hypotheses, students who reported lower levels of math anxiety who were assigned to the ER intervention had increased math grades compared to those assigned to the SS intervention. For individuals low in math anxiety, we speculate that perhaps the improved math performance observed in the ER group is attributed to the idea that cognitive reappraisal is a working memory-intensive strategy. The ability to look at an emotional situation through reframing or rethinking requires a lot of working-memory-intensive thoughts^37^. This emotion regulation strategy is effortful, and may be difficult to implement, especially when cognitive resources are already compromised by anxiety, or when learning a difficult new math skill. Because low math-anxious individuals may have had increased capacity for these emotion regulation strategies, this is a plausible explanation for why the ER intervention resulted in improved grade performance for low math-anxious individuals relative to the SS group.

This result conflicts with previous work on cognitive reappraisal and math anxiety done in a lab setting, but we must consider how context impacts cognitive capacity to engage in reappraisal^9,10^. These previous studies suggest that more highly math-anxious individuals are able to utilize a cognitive reappraisal strategy to improve math performance, suggesting that highly math-anxious individuals benefitted most from the cognitive reappraisal intervention in a lab setting. However, in these studies, participants were in a laboratory setting and were utilizing math skills that had previously been mastered (order of operations arithmetic problems). In other words, individuals may have been able to utilize a cognitive reappraisal strategy when they had the capacity to do so, such as in a low-stakes lab task, and performing a skill that was previously learned. Compared to the context of the current study, where students were in a scenario where their performance on the task resulted in real-world outcomes (grade performance), and where they were learning new information and skills, highly anxious participants in the previous lab studies may have had more cognitive capacity to utilize cognitive reappraisal skills. In the present study, individuals with lower math anxiety may have had a greater cognitive capacity to utilize cognitive reappraisal skills given the demands of the learning environment, and this may have resulted in improved math class performance relative to the study skills group.

Another possibility explaining the improved performance for highly math-anxious individuals in the SS intervention could be partially attributed to our use of a writing task in both intervention groups. Although past research suggests that the emotion-centered writing exercise was associated with improved performance for more anxious individuals^24,25,35^, it is possible that asking individuals in the SS intervention to write about the kinds of problems they thought would appear on the test would also result in a release of physiological arousal and decreased anxiety. Although the writing intervention for the SS group did not specifically focus on feelings or anxiety, writing out the kinds of problems they would expect to see on the test may have freed up working memory resources, helped students bring to mind the kinds of problems that they studied, thereby alleviating anxiety. In this way, students in the SS condition may have gotten a “double dose” of intervention, such that they may have had the benefit of both improving study skills and reducing anxiety through the writing intervention. Future studies could focus on the combined intervention of both study skills and anxiety support, as it’s possible that these may yield promising results for ameliorating the effects of math anxiety.

Indeed, another reason for the comparative success of the study skills intervention may be interrelation between anxiety and math performance. Over time, improved study skills and knowledge may have contributed to lessened feelings of anxiety and improved performance in a bidirectional relationship (Reciprocal theory^38^). Past research has suggested that poorer math performance is likely a driving factor in the development of math anxiety^39^, especially in younger populations in elementary school^40,41^. In addition to math achievement, additional factors and attitudes like math mindset^40^ and self-efficacy^42^ also contribute to the relation between math anxiety and math achievement. These previous results suggest that our study skills intervention may have capitalized on this reciprocal relation between math achievement and math anxiety, with improved study skills training contributing not only to improved math understanding and achievement, but also to reduced feelings of anxiety that may have further improved achievement. These two factors have a reciprocal relation that may gone from a “vicious cycle,” with negative emotion contributing to underachievement and vice versa, to a “virtuous cycle” where increased achievement and understanding may have a reciprocal relation with decreased anxiety. Although the present study is not well-suited to explore the longitudinal reciprocal relations between achievement and anxiety, we hope that future studies will explore the effects of these longitudinal relationships as future interventions are implemented.

This analysis and these studies also had some important limitations. For example, as in all studies that are implemented in a real-world educational environment, there are variations between the two samples of students, resulting in very different educational environments, and variation in the availability of identical measures across schools (i.e., variation in the assignments included in grade outcomes, timing and frequency of assessments, etc.). By using random effects in our models, we have attempted to control for some of this variability. If anything, these variations likely would have weakened the effects we observed of the SS intervention. Instead, whether the schools are analyzed in a combined sample or separately (See Supplementary Material), our results consistently illustrate that for more highly anxious students, the study skills intervention resulted in improved math class grades. Finally, although the current sample sizes limited the inclusion of a third, no-intervention control group within the same cohort, further investigations using similar intervention techniques should include a no-contact/waitlist control group in order to bolster the conclusions that these interventions increase mathematics grades. In the present study we addressed this concern by matching groups on prior achievement and performing within-subjects comparisons across academic terms, but future research would benefit from the addition of such a control group as well.

In summary, the results of these experiments suggest that the study skills intervention is a promising technique for reversing the effects of math anxiety on academic performance and increasing math grades. Improvements in study techniques, especially the frequency with which students use self-testing to learn and review material, likely encourages students to overcome their tendencies to avoid mathematics. Especially for highly anxious students in the study skills intervention, this process ameliorates the performance deficits associated with anxiety. These results suggest that interventions targeting study techniques have important implications for students who struggle with anxiety. Empowering students to improve their strategies for learning may decrease the deficits caused by anxiety in the classroom, encouraging students to excel.

## Method

### Participants

Participants in this study were recruited from two different school districts in geographically distinct regions of the US. School 1 was a small high school in rural New England. Classes were taught along different timescales: including semester-long, and year-long courses. At School 1, all students enrolled in these classes were invited to participate and parents were asked to opt-out of the study if they chose not to participate (parents were sent a letter informing of the study prior to the start of the study). The local superintendent, school administration, and Dartmouth Committee for the Protection of Human Subjects (CPHS #28333) approved these procedures that a written informed consent was not needed and that an opt-out sampling protocol was approved. No parents opted-out of the study. All students provided verbal assent to complete study procedures. One-hundred-nine adolescent participants were recruited for the study from their math classes (algebra I and II, geometry) taught by two instructors. From this sample, two students opted-out of the surveys, and 16 students had incomplete survey or grade data due to absences or technical difficulties with the online surveys. From the overall sample of *N* = 91 across six classes, students were between the ages of 13 and 18 (*M*_*age*_ = 15.34, *SD*_*age*_ = 1.05), and the sample was 60% female. Demographic information was provided by each school. For our analyses, we use the term “gender” to refer to masculine or feminine identity, as this encompasses the cultural and social context of gender roles, and our study does not refer to any biological measures of sex characteristics. The researchers realize there are a variety of identities that can be encompassed by gender identity, but for the purposes of these analyses, the researchers use the binary terms “male” and “female” to refer to the participants’ gender identity as this was the information provided by each school. For additional information about this sample, please see the Supplementary Material.

At School 2, approximately 272 students from a diverse high school in the mid-Atlantic region were invited to participate in this study, and parents provided a signed consent form for their student to opt-in to enroll in the study. Students were enrolled in Algebra I, Algebra II, and Algebra II honors classes, and were recruited from 13 year-long classes taught by six instructors. Out of this sample, 167 students had parents who provided a signed informed consent in order to participate in the study (59% response rate, 6 students not enrolled because they were enrolled in a different math class). In addition to the consent provided by parents, all students gave verbal assent. A total of 156 students were included in the dataset for analysis after an additional 5 students were excluded for incomplete grade information (*N* = 156, 53% female, *M*_*age*_ = 15.46, *SD*_*age*_ = .92, *Range*_*age*_ = 14–19; *n* = 21 Algebra I students, 43% female; *n* = 100 Algebra II students, 58% female; *n* = 34 Algebra II honors students, 44% female). For more information about the demographics of this sample, please see the Supplementary Material. Supplementary materials, an appendix of materials, and preprint versions of this manuscript are available through the Open Science Framework through PsyArXiv: https://osf.io/43q6y/.

All procedures were approved by the Dartmouth College Committee for the Protection of Human Subjects and each local high school’s administration. This study and its analyses were not preregistered. Students enrolled at School 2 did not receive monetary compensation for this study in accordance with school district regulations. At School 1, participants were entered in a gift card raffle by participating in follow-up surveys. The Authors declare no competing financial or non-financial interests.

### Design

Across both studies, all students were pseudo-randomly assigned to an intervention strategy group. To account for random effects created by class subject or teacher, each class was split in half, and half of the participating students in each class was assigned to each intervention group. Assignment to intervention groups were counterbalanced for gender, previous grade performance (School 2) and/or GPA and/or standardized test performance (School 1). Half of the students were assigned to an intervention technique focused on improving study skills (SS), half were assigned to an intervention technique focused on emotion regulation (ER) using cognitive reappraisal. In this way, we were able to establish that even within each class, the intervention groups would be relatively balanced in terms of gender composition and academic performance before the intervention was introduced.

This procedure of splitting each class in half also contributed to the decision to use two active intervention conditions in the study, instead of using a no-contact or waitlist control. Because classes needed to be split in half, this resulted in smaller groups within each class, and inclusion of an additional group would have further decreased the numbers of students assigned to each group, limiting our ability to draw conclusions about the impact of the interventions. Because no intervention materials were introduced during the first semester, we consider the first semester to be a within-subject control condition, as grades during the previous term or previous class could not have been affected by the assigned intervention. Because we were able to use a within-subject control for each students’ math performance, the researchers wanted to introduce two strategies that were both likely to have a positive impact on grade performance.

**Fig. 4:**
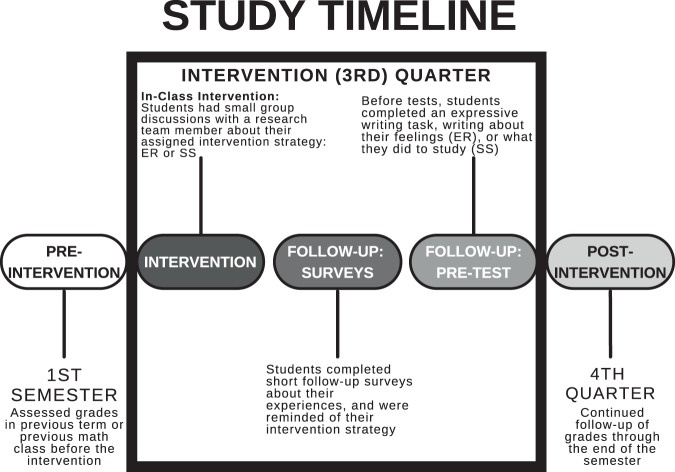
Study timeline across first and second semester. During first semester, no interventions were introduced, and we utilized grades during this period as within-subject control data. During second semester, an intervention was administered as a 20 min structured small-group discussion with a member of the research team during a class period. Each class was split in half and pseudo-randomly assigned to an Emotion Regulation (ER) Intervention group or Study Skills (SS) Intervention group. Groups were followed throughout the third quarter, with additional follow-up in the form of short surveys, reminders, and expressive writing tasks administered before major tests. During the 4th quarter, grades were recorded, and students received 2 brief survey prompts/reminders to determine how they implemented their intervention, but experimenters did not provide in-person sessions.

The intervention techniques were introduced during the second semester, with the main in-class intervention introduced at the beginning of the third quarter, with additional follow-up throughout the third quarter, and longitudinal follow-up with grades throughout the fourth quarter (Fig. 4). The main intervention was introduced in a short in-classroom session where students were split into small groups (approximately 2–10 students depending on the size of the class) based on assigned intervention strategy. Within each small group, students spent 20–30 min working on a worksheet in a structured discussion with a study team member who led the discussion. The interventions were designed to be personally relevant to the students, to encourage the student to consider how the assigned technique might be interesting or important to improving the way they react to anxiety in academic situations (Emotion Regulation Intervention Strategy) or improving their experience with different techniques designed to encourage students to study more efficiently (Study Skills Intervention Strategy). Continuing to observe grades and follow-up throughout second semester allowed us to observe the intervention over a longer timescale.

### Intervention strategies

Students were assigned to either an Emotion Regulation Intervention or a Study Skills Intervention. Both interventions were designed to be introduced in such a way that students saw their relevance to their daily lives, and could think about specific personal situations where they might have struggled with academic skills in the past, and how the intervention strategy could potentially help them in scenarios when students encountered academic challenges. In these structured discussions (20–30 min), the research team member asked students to explain how they would use the assigned strategy, and why it was useful. If students produced responses that were off-topic or incorrect, the research team member would redirect the response. Please see the Supplementary Material for an Appendix of in-class handouts.

The ER intervention was focused on how to use cognitive reappraisal in academic settings such as math class. The ER intervention focused on using an emotional *distancing* strategy encouraging the students to view the situation from a more objective perspective (“Imagine you’re explaining the problem to your best friend”). The ER intervention also introduced a *reframing* technique that aims to change the cognitive appraisals associated with a stress response, instead focusing on the possible positive associations with increased physiological arousal (“Think about the situation as a challenge rather than an obstacle,” “Stress may help you perform better, use these feelings to help you focus and overcome this challenge;” [33, 34]). This strategy was intended to allow students to reframe their reactions to math problems by changing their perspective and focusing on a mindset that would encourage them to approach mathematics, regulate their reactions, and face the problems at hand.

The SS intervention focused on two study skills: spaced studying^16^ and retrieval practice^4,11,43,44^. In spaced studying, students were encouraged to avoid cramming, and set aside time to review key information on a regular basis. In retrieval practice, students were encouraged to practice bringing information to mind by using self-testing, such as doing practice problems, taking practice quizzes or tests, and making flashcards as effective ways to study for their math class. As in the ER intervention, the discussion was structured to focus on what students were already doing to study, where they were encountering problems, and where they could change their behaviors to focus on building study habits that were supported by spaced studying and retrieval practice to make these behaviors more personally-relevant to each student.

#### Surveys

After completing the small group discussions, students completed a series of questionnaires. These survey measures were meant to assess a baseline measure of various types of academic anxiety. Students completed standardized measures of test anxiety (Test Anxiety Inventory, TAI^45^, math anxiety (Math Anxiety Rating Scale, MARS^46^, trait anxiety (State-Trait Anxiety Inventory, STAI^47^, and the Emotion Regulation Questionnaire (ERQ^48^ Students also completed the Academic Anxiety Inventory (AAI), a self-report measure designed to test math anxiety, as well as anxiety related to tests, science, writing, and trait levels of anxious emotion^49^.

### Follow-up intervention activities

In addition to the in-class structured discussion, students received reminders to implement their assigned intervention strategy throughout the term. Before an exam in their math class, students were asked to write about their assigned intervention strategy, similar to previous research on expressive writing interventions^24,25^. In the Emotion Regulation Intervention, students were asked to write about the thoughts and feelings they would experience while taking the upcoming test (for example: “please write as openly as possible about some of the thoughts and feelings you might experience on your upcoming test”), and were also asked to write about what reappraisal strategies they would use during the test to rethink or reframe their experience. In the Study Skills Intervention, students were asked what kinds of math problems they thought would appear on the upcoming test, and what strategies they might use to solve these problems on the test. Students were directed to write a few sentences in response to each question, and responses were collected on a pencil-and-paper worksheet. For school 1, students completed this writing activity before a midterm exam given during the third quarter. For school 2, students completed the writing activity during summary unit tests completed throughout second semester (approximately 4 tests).

In addition to the pre-test writing activities, students were also expected to fill out short questionnaires during the third quarter, and continuing with lower frequency during fourth quarter. Students completed a 6-question survey that involved answering brief questions about feelings of anxiety, understanding, confidence, as well as how frequently students had used aspects of the intervention techniques in the past few days. This short questionnaire was repeated several times throughout the semester over the course of the intervention. In School 1, the researchers received feedback that some students had difficulty remembering the material from their assigned strategy. Subsequently, for School 2 these short surveys were combined with additional reminders about the assigned intervention strategy (See Supplementary Material for implementation differences between schools). In order to remind students about their assigned technique, students were given short reminders to identify the correct intervention technique, and were asked to write down ways they could use their assigned technique while working on assignments for math class. Additional reminders were administered during class time or during study hall periods (see Supplementary Materials for examples). In the present manuscript, we focused on grade performance, and analyses evaluating additional survey outcomes are included in the Supplementary Material.

### Grades

In this study, our main outcome measures were the grades given in these real-world math classes. However, for each school, grade composition differed slightly. For School 1, we analyzed quarter grades in the third and fourth quarter of the school year, which included tests (including the midterm exam which included the test-related intervention activities), homework, quizzes, and other miscellaneous assignments within the 8–10 week quarter. Previous grades were also provided to account for previous mathematics performance, which included grades within the same course for year-long courses, and overall grade for the previous mathematics course for semester-long courses. For School 2, we focused on analyzing grades from classwork, homework and quizzes, as these grades only reflected performance during the quarters where the intervention strategies had been introduced, as cumulative quarter grades also included previous course performance from the first semester. For year-long classes, the previous math performance was drawn from previous quarter grades. For more details about grade information for each sample, please see Supplementary Material.

### Reporting summary

Further information on research design is available in the Nature Research Reporting Summary linked to this article.

### Supplementary information

Supplementary Material

Reporting summary Checklist

## Data Availability

Some materials associated with this project will be shared in the Open Science Framework online repository (https://osf.io/43q6y/). These data include educational data from underage minors. Sharing of these data is restricted based on FERPA regulations and research agreements with the participating local schools. Due to the nature of the data, deidentified data and code will be shared by request from the authors, but complete de-identified data will not be shared in an online repository due to privacy restrictions and the sensitive nature of the educational data. Interested parties can contact the corresponding author, Rachel Pizzie (Rachel.pizzie@gallaudet.edu) to request access to the data and should receive a response to requests within two weeks. Interested parties will be asked to describe how they will ensure privacy and confidentiality of the data and maintain the security of the data. Interested parties must agree not to share these data beyond the parties included in the request, and do not have permission to publish the data themselves.

## Code availability

Corresponding to the sensitive nature of the data, code for this project will be shared by request from the authors, but will not be shared in an online repository due to privacy restrictions and the sensitive nature of the educational data.
