# Supplementary material for: Strategies for remediating the impact of math anxiety on high school math performance
Source: NPJ Sci Learn. 2023 Oct 2;8:44. doi: 10.1038/s41539-023-00188-5 (PMC10543627; doi:10.1038/s41539-023-00188-5)
Supplement: Supplementary file 1 — Supplementary Material [file 41539_2023_188_MOESM1_ESM.pdf]

## Supplementary Information

### Supplementary Notes 1

In School 1, we sought to test both intervention techniques, one targeting emotion regulation (ER), and the other focusing on utilizing better study skills (SS) in high school mathematics classrooms. Both intervention techniques were designed to include small group discussions with high school students at the beginning of the second semester, then students were followed with questionnaires throughout the quarter, and both groups completed a short writing task immediately before the midterm exam (Ramirez & Beilock, 2011). Both interventions were designed to overcome anxiety and avoidant behaviors characteristic of math anxiety, though in different ways. The SS intervention focused on creating more efficient study strategies, both in organizing the time needed to study, as well as reducing avoidant behaviors by encouraging anxious students to gain more practice with the kinds of problems they were learning about.

The ER intervention provides an adaptive strategy to reduce feelings of anxiety by encouraging students to reframe or rethink the situation that was causing these negative feelings. We hypothesized that the SS intervention would positively affect grade performance for all students, including those who were low in math anxiety. We also tested whether this approach was equally effective for highly anxious students, whether students of all anxiety levels adhered to the SS and ER intervention strategies, and whether habitual use of emotion regulation strategies (not directed by the experimenters) would predict success with this intervention. For the ER intervention, we did not expect non-anxious students to show large increases in performance, as anxiety does not typically interfere with their performance. However, we hypothesized that the ER intervention would be advantageous for anxious students, improving both grades and attitudes toward mathematics. Again, for this group we also measured adherence

as well as habitual use of ER strategies. Both intervention strategies were designed to be easy and cost-free to implement in a classroom setting, aiding students by introducing flexible, intuitive strategies that could reduce avoidance of mathematics, and reduce the decline in performance associated with anxiety, thus encouraging students to reach their full potential.

**Supplementary Methods 1**

*School 1 Participants.* One-hundred-nine adolescent participants were recruited for the study from their math classes (algebra I and II, geometry) in a rural high school in New England. Nine students were enrolled in multiple mathematics courses, and one student was enrolled in two sections of the same course. Parents and guardians were informed about study participation and were given the opportunity for their student to opt-out of any procedures; no parents opted-out of the study. All students were read an assent statement outlining the study procedures and their rights and responsibilities as research participants, and provided verbal assent to complete study procedures. All procedures were approved by the Dartmouth College Committee for the Protection of Human Subjects and the local high school administration. From this sample, 2 students opted-out of the surveys, and 16 students had incomplete survey or grade data due to absences or technical difficulties with the online surveys. The resulting sample included 91 students enrolled in the study across six classes. Students were between the ages of 13 and 18 ($M_{age}$ = 15.34, $SD_{age}$ = 1.05), and the sample was 60% female. Further demographic data such as race and measures of SES were not provided for this specific sample, but based on data from the 2014-2015 school year, the school is 99% white, 1% black, 1% Hispanic, and 41% of students are rated as economically disadvantaged (37% free lunch, 4% reduced-price lunch), and have 26% of students who are rated as proficient in mathematics (US News and World Report). Because student performance in each class is rated independently (i.e., the student's grade in one

course does not affect the grade in the other course), students enrolled in multiple courses were treated as independent subjects in these analyses (*n* = 99 samples).

Students were pseudo-randomly assigned to group on the basis of gender and previous GPA or standardized math test performance (students in 9th grade did not have a previous GPA so standardized test scores were used), so that each group was balanced in gender distribution and a measure of previous academic performance (ER Group: 60.8% female, $M_{GPA}$ = 2.95; SS Group: 58.33% female, $M_{GPA}$ = 3.07). Each class was split in half, and approximately one half was assigned to the study skills intervention, and one half assigned to the emotion regulation intervention. Students who were taking multiple classes were assigned to the same intervention strategy in each class[1].

*School 1 Design.* At the beginning of the intervention quarter (Supplementary Figure 1), students were instructed in one of two interventions, and completed a series of surveys to assess individual differences in anxiety. The intervention took place during a single quarter of the school year, but examining grades during the quarter after the intervention allowed observation of the potential effects of the intervention over a longer timescale when students were no longer being actively instructed to implement either strategy. Student grades were provided for previous mathematics performance (within the same course for year-long courses, and for the previous mathematics course for semester-long courses), midterm exam, intervention quarter grades (includes homework, quizzes, etc.), and final exam and final quarter grades. To analyze grade performance, we utilized quarter grades, which included tests (including the midterm exam that included an expressive writing intervention), homework, quizzes, and other miscellaneous grades for each quarter (approximately 8-10 weeks). The intervention quarter was the first quarter in

[1] All analyses were also completed excluding the students who were enrolled in multiple classes. The effects reported here are similar when these participants (*n* = 9) are excluded.

second semester (3rd quarter), and students' grade performance was also reported for the last quarter of the school year (4th quarter) to evaluate how grade performance was influenced after the intervention to examine longitudinal effects.

*School 1 Intervention Strategies.* During class time, students were broken into small groups to learn about their assigned intervention from a researcher. Each intervention was structured to be completed in approximately 20 minutes, and involved a semi-structured discussion between students and an experimenter, as well as completing a worksheet to reinforce the information discussed. The SS intervention included instruction and discussion focused on time management, including scheduling time to study and spacing out study sessions. The SS intervention also focused on effectively learning content, including interleaving topics and using self-testing to focus on completing practice problems.

The ER intervention focused on cognitive reappraisal, specifically focusing on rethinking a negative situation in order to change one's negative or anxious emotional reaction. Students were instructed to think about distancing themselves from feelings of anxiety in generally stressful situations, as well as using this strategy in math class ("Think about explaining this problem to your best friend." "Think about your teacher explaining this problem to you, what would she tell you to do first?").

In both groups, students were asked to explain how they would use this strategy, why it was useful, and student responses were redirected by the experimenter if they produced responses that were off-topic in order to encourage understanding of the assigned intervention. Both strategies were designed so that students would be interested in the material because it was self-relevant, and motivated to incorporate these strategies themselves, in the hope that students who were self-motivated would be more likely to actually utilize these intervention strategies on

a regular basis.

*School 1 Surveys.* After completing the small group discussions, students completed a series of questionnaires (surveys were presented using Qualtrics, www.qualtrics.com). Students completed measures of test anxiety (Test Anxiety Inventory, TAI; (Spielberger, 2009), math anxiety (Math Anxiety Rating Scale, MARS; (Suinn & Winston, 2003), trait anxiety (State-Trait Anxiety Inventory, STAI; (Spielberger, 2010), and the Emotion Regulation Questionnaire (ERQ; (Gross & John, 2003). Students also completed the Academic Anxiety Inventory (AAI), a self-report measure designed to test math anxiety, as well as anxiety related to tests, science, writing, and trait levels of anxious emotion (Pizzie & Kraemer, 2017; 2015). Students also completed a 6-question survey that involved answering brief questions about feelings of anxiety, understanding, confidence, as well as how frequently students had used aspects of the intervention techniques in the past few days. This short experience-sampling questionnaire was repeated several times throughout the quarter in order to assess how these feelings change over the course of the intervention. Students were compensated for completing surveys by entries into a raffle to win gift cards. Students were followed with these short surveys until the midterm exam approximately 8 weeks later at the end of the intervention quarter.

*School 1 Midterm Intervention.* Immediately before the midterm exam, students in each group completed a short writing exercise similar to the procedure developed by (Ramirez & Beilock, 2011). Each intervention lasted 7 minutes and was designed to implement the skills utilized by each intervention strategy, and was administered by an experimenter. Students in the SS intervention were instructed to "write down some of the kinds of math problems you think will appear on your test and some of the strategies you might use to solve them." This intervention was designed to encourage students to report the results of engaging in practice

problems, additionally reducing anxiety by listing their expectations for the kinds of challenges they would face and invoking previous strategies that were advantageous while studying. The ER intervention instructed students to “write down some of the ways you could use 'reappraisal’ (rethinking your perspective) on your test.” This intervention prepared students to engage in emotion regulation should they begin to feel anxious during the course of the midterm exam. Students were also asked again to complete the short experience-sampling questionnaire to assess feelings and opinions regarding math as well as frequency of use of each of the techniques utilized in the interventions. After these writing exercises and questionnaires were completed and returned to the proctors, students completed their midterm exam as a part of normal class procedures.

## Supplementary Discussion 1

All analyses were completed using a combination of R statistics (R Core Team, 2013) and SPSS (IBM Corporation, 2012).

*School 1 Grades.* We analyzed overall grades from the quarter during the intervention (intervention quarter) as well as the subsequent quarter (final quarter). We used a mixed-design ANOVA to evaluate how overall grade performance changed during and after the intervention (within-subject) for each intervention group (between-subjects). There was no significant main effect of intervention group, $p > .20$. There was a significant main effect of quarter, $F(1,93) = 8.36$, $p = .005$, $\eta_p^2 = .083$, such that quarter grades were higher overall during the intervention. There was a significant interaction between quarter and intervention group, $F(1,93) = 7.07$, $p = .009$, $\eta_p^2 = .071$ (Supplementary Figure 2). In the intervention quarter, the SS intervention group ($M = 78.83$, $SD = 14.39$) showed higher grades compared to the ER intervention group ($M = 72.47$, $SD = 20.64$), $F(1, 93) = 2.994$, $p = .087$, whereas these groups were not significantly

different from one another in the final quarter, $p > .3$. Grades in the SS intervention group were significantly higher during the intervention quarter ($M = 78.83$, $SD = 14.39$) compared to the post-intervention quarter ($M = 72.22$, $SD = 17.13$), $F(1,93) = 14.93$, $p < .001$, and grades did not differ for the ER intervention group across quarters, $p > .3$.

In order to determine whether this difference in groups was determined by other factors, including previous course performance, or what can be expected by normal grade performance throughout the year, we followed up on these group analyses across the intervention and post-intervention quarters. To account for the effect of previous performance, we examined these effects in a subset of participants enrolled in year-long classes ($n = 67$), and used performance from the previous semester to control for previous class performance. We used linear mixed models first to replicate our analysis in a subset of students enrolled in year-long classes, using a fixed factor for each quarter (Significant main effect of quarter: $\chi^2$ (1) = 5.23, $p = .02$), a fixed factor for intervention group (Nonsignificant main effect of group: $\chi^2$ (1) = .91, $p = .34$), and random effects accounting for each individual. We again find a significant interaction between quarter and intervention group, $\chi^2$ (1) = 3.85, $p = .049$. We then added random effects accounting for performance during the previous semester (before intervention groups were introduced) to this model. Controlling for previous performance, we again find a significant main effect of quarter, $\chi^2$ (1) = 5.11, $p = .02$, no significant main effect of group, $\chi^2$ (1) = .71, $p = .39$, and a significant interaction between quarter and intervention group, $\chi^2$ (1) = 4.26, $p = .039$. These results illustrate that even controlling for previous performance in the course, we still find that the SS intervention group shows elevated performance during the intervention quarter compared to the ER intervention group.

We also compared performance trends to an additional control group of students from a

previous year enrolled in similar algebra and geometry courses at the same school with the same instructors. For this control group, we compared grades taken midway through the year and compared these to the intervention quarter, to final term grade performance and compared these to the post-intervention quarter (all grades represent a composite of homework, quizzes, tests, and class participation). Here we used a linear model to compare difference scores between the midterm/intervention quarter and the final quarter between the SS intervention group, the ER group, and the previous year's group (no contact control group). Here we find that the SS intervention showed a significantly greater grade increases in the intervention quarter compared to the final quarter ($M$ = 5.82 points greater in intervention quarter, $SE$ = 1.38), whereas the ER intervention showed very little difference between intervention and final quarters ($M$ = .24 points, $SE$ = 1.33), and the previous year's group also showed very little difference between terms ($M$ = .10, $SE$ = 1.72), $F(2, 121) = 5.31$, $p = .006$. These results illustrate that the SS intervention resulted in grade increases during the intervention quarter, above and beyond what can be explained by normal fluctuations in grade performance.

*School 1 Demographic Factors.* In order to further examine the influences of other factors on grade performance, we compared the influence of various classroom factors[2] that may have influenced results during the intervention quarter. Though previous investigations into the impact of stereotype threat on mathematics performance have found that female students underperform in math when subjected to stereotype threat (Johns, Inzlicht, & Schmader, 2008; Krendl, Richeson, Kelley, & Heatherton, 2008; Schmader & Johns, 2003), in our sample, we find that female students ($M_{female}$ = 79.47%) consistently outperform their male counterparts ($M_{male}$ = 70.15%) during the intervention quarter, $t(79.19) = 2.51$, $p$ =.014. This result was also

[2] For discussion of the influence of different instructors on grade outcomes, see Supplementary Information.

replicated during the final quarter grades, $t(81.53) = 2.83$, $p = .005$. That female students excel in grade performance is consistent with recent trends in educational research, including studies that examined large cohorts of students schooled in the 1980s-2000s (Deary, Strand, Smith, & Fernandes, 2007; Fergusson & Horwood, 1997; Wirt et al., 2000). Gender did not interact with intervention group to influence grade outcomes, nor did it interact with group and individual differences in anxiety, all *p*s > .2. Self-reported math anxiety (AAI-Math scores) did not differ by gender, $t(91.91) = 1.07$, $p = .29$. Though gender does influence grade performance, giving the advantage to female students, the results that we observe in terms of the intervention cannot be attributed to gender effects or stereotype threat.

*School 1 Individual Differences in Anxiety.* In order to determine how the two interventions interact with students' anxiety levels to influence math grades, we evaluated academic performance as a function of several factors: the assigned intervention and individual differences in test anxiety, trait anxiety, and math anxiety. To predict academic performance (quarter grades), we used separate linear models for each type of anxiety, including scores on anxiety measures and assigned intervention group as between-subject factors.

First, we examined how test anxiety (TAI scores) interacts with intervention strategy (SS and ER) to predict math course grades, using linear regression with TAI and intervention group as between-subject factors (Supplementary Figure 3). There was a significant main effect of group on intervention quarter grades, $F(1,90) = 4.97$, $p = .03$, such that intervention quarter grades were higher for the SS group than the ER group. There was also a significant main effect of test anxiety on intervention quarter grades, $F(1,90) = 4.85$, $p = .03$. This main effect of anxiety replicates previous research (Cassady & Johnson, 2002; Culler & Holahan, 1980; Spielberger, 2009), indicating a negative correlation overall between anxiety and performance. There was

also a significant interaction between intervention group and test anxiety, $F(1,90) = 5.69$, $p = .02$ (Supplementary Figure 3a), indicating the greater effectiveness of the SS strategy. Students in the ER group showed the commonly-observed anxiety-related performance decline (Agarwal, D'Antonio, Roediger, McDermott, & McDaniel, 2014a; Messineo et al., 2015; Ramirez & Beilock, 2011), such that increased anxiety was associated with lower grades. However, students in the SS group did not show this negative association with anxiety (Supplementary Figure 3c), and increased anxiety was not associated with a decline in grade performance.

We observed a similar result when analyzing the relationship between intervention group and trait anxiety (STAI; Supplementary Figure 3b). There was a main effect of intervention group, such that students in the SS group had higher intervention quarter grades, $F(1,90) = 4.91$, $p = .03$. There was a main effect of trait anxiety on intervention quarter grades, $F(1, 90) = 5.05$, $p = .03$, such that the more anxious students had lower grades. Similar to the effects observed for test anxiety, there was a significant interaction between intervention group and trait anxiety on intervention quarter grades, $F(1, 90) = 4.28$, $p = .04$. Again, while the ER group showed a decline in grades associated with increased trait anxiety, the SS group showed no relationship between trait anxiety and performance, showing a significant increase in performance for those highest in anxiety (Supplementary Figure 3d).

When examining the fourth quarter, the effects of the intervention did not persist: we no longer observed a significant interaction between anxiety and group on final quarter grades, although the main effect of anxiety on performance remained significant for both measures of anxiety. For test anxiety, there was no main effect of group, $p > .3$ on final quarter grades. The interaction between group and test anxiety was no longer significant when comparing final quarter grades, $F(1,90) = 2.03$, $p = .15$. However, there was a main effect of test anxiety on final

quarter grades, such that increased anxiety was associated with decreased performance, $F(1, 90) = 5.31$, $p = .02$. We observed an identical pattern in trait anxiety, such that there was no main effect of group, $F(1, 90) = .65$, $p > .3$, and the interaction between trait anxiety and group was not significant for final quarter grades, $F(1,90) = 2.82$, $p = .09$. Again, we observed a significant main effect of trait anxiety on final grade performance, $F(1, 90) = 4.55$, $p = .04$, indicating an overall negative trend for performance as anxiety increases. When we examined final quarter grades after the intervention, we no longer observed the protective effects of the SS condition for those high in anxiety, and we observed a return of the negative effects of anxiety on grade performance across both groups.

For both trait and test anxiety, students assigned to the ER intervention group seemed to be unaffected by the intervention, showing characteristic anxiety-related declines in performance that are also consistent with previous literature on anxiety in classrooms (Ashcraft, 2002; Beilock & Maloney, 2015; Cassady & Johnson, 2002). However, in the SS condition, it seems that improving study skills bolstered mathematics performance against the negative effects of anxiety, such that even at similarly high levels of anxiety, we no longer observed any decline in performance during the intervention quarter. Although our results show that the effects of the SS intervention did not persist beyond the intervention quarter, the within-subject comparison between quarters clearly indicates that the students in the SS group benefitted from the intervention while they regularly completed surveys about their study skills. That we observed these positive effects in the SS group and not the ER group suggests that encouraging students to gain more exposure to mathematics by utilizing effective strategies such as self-testing can have significant effects on grade performance.

To examine effects of math anxiety on quarter grades, we entered our math anxiety

measures (MARS scores and AAI-Math scores) into separate linear regression models along with intervention group as between-subject variables. There were no main effects or interactions for the MARS, and although there was a main effect of AAI-Math subscale on intervention quarter grades (negative association, $F(1,91) = 4.54$, $p = .035$), there were no other significant main effects or interactions for any of the AAI subscales (AAI-Test, AAI-Trait, AAI-Science, AAI-Writing), all *p*s > .05.

*School 1 Emotion Regulation Strategies.* Although we utilized instruction about cognitive reappraisal as an intervention strategy targeted to ameliorate feelings of math anxiety, emotion regulation strategies can also be conceptualized as habitual, or dispositional tendencies. As such, we additionally chose to explore dispositional tendencies to use cognitive reappraisal or expressive suppression using subscales of the Emotion Regulation Questionnaire (ERQ; (Gross & John, 2003).[3] In this way, we were able to examine how our instructed strategies might interact with students' intuitive use of emotion regulation. Using a new regression model, we examined how individual differences in each self-report scale interact with intervention group to influence mathematics performance in terms of intervention quarter grades. We found a significant interaction between intervention group and suppression, and a similar interaction for the reappraisal subscale of the ERQ (Supplementary Figure 4), described in detail below.

Using intervention quarter grades as an outcome measure we observed interesting interactions between intervention group and both ERQ subscales (Supplementary Figure 4). We observed a significant pattern of results when examining this interaction using the ERQ-ES subscale, which indicates habitual use of expressive suppression. We found a trending main

[3]As with previous investigations using the ERQ, we find that the subscales are not correlated with one another, $p = .3$. However, as expected, reappraisal subscales were inversely correlated with measures of anxiety (AAI-trait: $r(97) = -.270$, $p = .007$), and the suppression subscale was positively associated with other measures of anxiety (AAI-trait: $r(97) = .36$, $p < .001$).

effect of group, $F(1,91) = 3.21$, $p = .08$, and no main effect of ERQ-ES, $p > .2$. There was a significant interaction between Group and ERQ-ES, $F(1,91) = 7.02$, $p = .01$ (Supplementary Figure 4a), such that increased habitual suppression was associated with a decline in performance for the ER intervention group, but increased habitual suppression was associated with better intervention quarter grade performance for the SS strategy group.

For the cognitive reappraisal subscale of the ERQ (ERQ-CR), we again found a trending main effect of group, F(1,91) = 2.98, p = .09, and no main effect of ERQ subscale. For the reappraisal subscale, there was not a significant interaction between intervention group and ERQ-CR, $F(1,91) = 1.06$, $p = .31$ (Supplementary Figure 4b). Though the interaction is not statistically significant, increased reappraisal in the SS intervention is associated with better grade performance during the intervention quarter. In the ER intervention group, there was no relationship between reappraisal and grade performance.

Thus, the combination of study skills with an increased tendency to be aware of and regulate one's own emotions was associated with the best performance outcomes, although more research is needed to determine how reliable is this finding and what circumstances might strengthen this effect. Specifically, students in the ER intervention group performed best when they reported low levels of habitual suppression, which is perhaps due to decreased experience of anxiety compared to those who report using suppression on a regular basis in order to cope with stress. These results indicate an important role for emotion regulation in academic anxiety, and suggest that encouraging habitual practices of emotion regulation combined with improved practices for studying and learning mathematics results in better mathematics performance.

*School 1 Adherence: Writing Samples in Midterm Intervention.* We also explored the written responses given by students during the writing exercise given before the midterm exam.

We recorded 89 responses from these students (some students were absent on midterm test day). In this way, we were able to gain insight into whether students remembered the instructions they were given for their selected intervention technique. Students in the reappraisal condition were asked how they would use reappraisal (thinking about the problem from a different perspective), and students in the study skills condition were asked to list the kinds of problems they thought they would see on the test, and techniques to solve them. For the ER intervention, some characteristic examples of students who correctly interpreted the intervention strategy included "take deep breaths, look at problems from a different perspective, focus, stay on task, stay calm," and "On my test I could rethink my perspective to see and make sure I've donewhat my teacher wants/how she wants me to do it, and try to make sure I've done it correctly before turning it in."

Responses in the SS intervention condition were frequently similar to the following examples: "We will probably be asked about polygons, quadrilaterals, triangles, perimeter, proofs, ect.. I will use the formulas and as many of the postulates I can remember to solve them," and "The problems that will appear on my test will involve logs, exponential equations and I will solve them to the best of my ability and use my property sheet and what I learned in class. If it's a word problem, I will make sure to write down all the usefull information and solve the problems." However, frequently, students provided responses that referenced helpful techniques for test-taking, but that were not specifically related to their assigned technique, such as "You could reword the problem. Move on and come back later as if it were a new problem. You could compare it to other math related tasks you know," or "Try and calm down and skip the hard problems and go back to them at the end. This way you can see everything you know done." Responses were rated on being related to the assigned technique, testing technique, or a combination of both. In the ER intervention group (46 responses, including 3 unrelated

responses, e.g., "I could not as well that'd be great"), approximately ~35% of students produced a response related to the ER technique, ~15% produced a response related to ER but also incorporating testing techniques, and ~43% produced responses related to testing techniques but not related to using reappraisal. In the study skills group (43 responses including 6 unrelated responses), ~62% of students produced a response related to the SS technique,  ~11% of students produced responses incorporating listing the problems and techniques but also test-taking techniques, and ~9% produced responses that related to testing techniques but not related to the SS prompt. Overall, these responses suggest that perhaps the students in the ER intervention had a difficult time remembering their instructed intervention technique, and defaulted to using familiar (and useful) test-taking strategies that were not directly related to emotion regulation.

*School 1 Adherence: Experience-Sampling Survey.* In addition to evaluating test grades and quarter grades, the interventions used in this study were also designed to target opinions about mathematics, and to encourage students to use better study habits and emotion regulation strategies. Students were given an experience-sampling survey in class immediately after learning about their assigned intervention strategy, at another time approximately halfway through the quarter, and immediately before the midterm exam.[4] Using this 6-question survey, we evaluated how feelings of confidence, lack of understanding, and math anxiety changed throughout the intervention quarter, comparing the scores across time within each subject, and examining whether these scores were affected by intervention group assignment using a mixed-design ANOVA. No significant interactions were found for self-reported measures of confidence, lack of understanding, or math anxiety, all *p*s > .3.

[4] Students were also encouraged to complete this 6-question survey online during the intervening weeks, although due to distribution difficulties and lack of interest, there were very few students who consistently completed the online surveys. These data are not included in this analysis.

We also examined the frequency with which students used behaviors associated with each intervention technique, and whether these frequencies differed for each intervention group using a mixed-design ANOVA. No significant interactions were found for frequency of self-reported emotion regulation (e.g., “Tried to think about problems from a different perspective”), or frequency of use of organizational habits (e.g., “Tried to use a schedule to organize my study habits”).

However, for self-testing, we found a significant main effect of time point on ratings of self-testing, $F(2,152) = 7.65$, p = .001, $\eta_p^2 = .09$, such that self-reports of self-testing frequency (e.g., “Tried to review information by testing myself and practicing problems”) increased over time from the beginning of the intervention up until the midterm exam (Supplementary Figure 5). Further we found a significant interaction between intervention group and self-testing frequency over the course of the intervention quarter, $F(2,152) = 4.20$, $p = .017$, $\eta_p^2 = .052$. Students in the SS intervention group reported using self-testing or using practice problems more frequently across the course of the third quarter. However, for students assigned to the ER intervention group, frequency of self-testing as a study strategy stayed the same across the course of the intervention quarter.

Students assigned to the SS intervention group reported utilizing the instructions provided in this intervention more frequently across the intervention quarter by using self-testing or completing practice problems. In contrast, students in the ER intervention group did not report an increase in using the assigned ER strategy (nor did they report a significant increase in using study strategies). This increased adherence in the SS intervention group may provide additional insight into the efficacy of this intervention in encouraging better performance. Even introducing the idea of self-testing in a 20-minute intervention resulted in increased use of this technique

after 8 weeks, and the SS intervention was also associated with better performance during this time period, further illustrating that this intervention results in promising behavioral changes and increases in performance in math class.

*Additional information, School 1: Demographic Effects on Grades.* We examined the effects of teacher on grade performance, comparing the two instructors in terms of quarter grade performance. To account for pre-existing differences between the two teachers and grading styles, students were pseudo-randomly assigned to condition with half of each class assigned to each intervention. GPA (pre- intervention) scores were used to ensure that each group was balanced in terms of pre-intervention performance. There are no differences between teachers in 2nd quarter (pre-intervention) grades, nor any interaction between teacher and group assignment on grades, all *p*s > .3. One instructor gave higher grades than the other across both the intervention quarter, $t(88.52) = 2.59$, $p =.01$, and final quarter, t(92.74) = 3.42, p = .0009. However, this same pattern was found on grades given during the first quarter (prior to the intervention), $t(64.413) = 4.99$, $p < .0001$, indicating that although these differences were not captured in the second quarter grades, the two instructors differed in grades assigned across the board.

Similarly, we find significant interactions between group and teacher for grades given during the intervention quarter, $F(1,91) = 5.93$, $p < .02$, and final quarter $F(1,91) = 4.63$, $p = .03$. However, these interactions are likely due to chance assignment of students to each group, such that we find similar patterns of mean differences between the intervention groups across the quarters, such that for all four quarters (See Supplementary Table 1), including prior to the assignment to the intervention, for instructor 1, we observe higher means for the SS intervention across all 4 quarters, and for instructor 2, the means between the intervention groups are

relatively similar or slightly favor the ER intervention. However, the interactions between group and instructor do not reach statistical significance during the first semester, *p*s > .3. As a result, the differences that we observe between groups and instructors are likely due to teacher and group differences that existed before assignment to the intervention, that may have been further exacerbated by participation in the interventions, given that we observe similar effects across the year, though these effects intensify during and after the intervention. Further, three-way interactions including, group, instructor and anxiety were evaluated to see if these instructor effects influenced the interactions between anxiety and intervention group. We observed no three-way interactions between instructor, group, and test or trait anxiety, p > .3. In other words, though we do observe instructor and group differences, these do not influence individual differences in anxiety and the impact anxiety has on performance in each intervention group.

As a result, for the remainder of the analyses, the researchers have decided to collapse across both instructors, given that the experimental design of this study split each class in half between each intervention group (and were equally distributed in terms of gender and GPA) and should keep the individual differences in instructors constant across grading periods. The researchers recognize that individual differences among instructors are a common feature of experiments in real-world classroom settings. That the rural school that participated in this study had only two instructors who taught high school math classes is an interesting feature of our particular sample, and may be instructive for future studies and interventions that should likely include samples of students in rural areas. Future investigations will hopefully include individual differences in instructors and other demographic factors so that their impact on classroom outcomes may be more effectively studied.

In School 1, we introduced two different strategies into a classroom setting with the goal

of improving mathematics understanding and performance, as well as reducing the negative effects of anxiety related to tests and math, as well as general feelings of anxiety. Overall, the results indicate that the intervention strategy focused on better study skills was the most advantageous. Students in the SS intervention group showed increased math performance during the intervention quarter, and this intervention reduced the negative impact of anxiety on grades. The positive effects associated with this intervention strategy are likely attributed to the adherence to the intervention itself. Students in the SS intervention group reported using self-testing more frequently over the course of the quarter leading up to their midterm exam, whereas we did not observe any self-reported changes in attitudes or frequency of emotion regulation strategies for the ER intervention group.

In contrast to habitual emotion regulation, the ER intervention did not have the desired effect on participants. One likely explanation for this null finding is that students did not understand or remember the intervention after the initial introduction session, as evidenced by low self-reported adherence. Whereas students in the SS intervention did report increases in self-testing, students in the ER group reported no change in the frequency of reappraisal throughout the intervention quarter, indicating that this intervention was not incorporated into students' behavior. A further indication that the ER strategy was not successfully adopted by students in the ER group comes from a qualitative evaluation of their writing intervention responses. During the writing intervention, students in the ER group wrote about skipping difficult problems and coming back to them, a technique that is not consistent with the instructions the students received to reappraise their anxiety. This lack of understanding of the intended intervention seems to have contributed to the null effect of the ER intervention, as evidenced by the persistent negative correlation between test anxiety and academic performance, which has consistently been

observed in the past (Culler & Holahan, 1980; Hembree, 1988; Messineo et al., 2015). For many students, learning about emotion regulation (especially as it relates to mathematics) was a novel concept, and students may have struggled to remember the specifics of using the intervention. In contrast to the null results found in the ER intervention used in this study, previous work has indicated that reappraisal can be an effective technique for reducing the negative effects of stress and test anxiety (Jamieson et al., 2016), influencing psychophysiological responses to stressors (Jamieson, Mendes, Blackstock, & Schmader, 2010; Jamieson, Nock, & Mendes, 2012). In the future, our intervention would be strengthened by improving students' understanding of this technique, utilizing different examples or phrasing, or perhaps by adding reminders and feedback about the intervention using appropriate examples throughout the course of the term.

In addition, there are some important limitations to consider before these results can be generalized to other populations. First, although Study 1 included almost 100 students in several different classes, the school student body utilized in this study is quite small and does not represent a racially or socioeconomically diverse environment. Similarly, this study only included two instructors, and idiosyncrasies in grading and assessment techniques could have influenced grade outcomes; the study would be strengthened by including a wider variety of instructors (see Supplementary Information).

Overall, the results of Study 1 introduce the SS intervention as a promising technique for improving math performance and ameliorating the negative relationship between increased anxiety and performance in math class. Further, we find that students who habitually use emotion regulation techniques may have the best outcomes when they are taught to incorporate techniques that improve their study skills.

---

## Supplementary Notes 2

In School 2, we sought to replicate our results from Study 1, while bolstering the reminders about both interventions throughout the intervention quarter. In giving additional reminders about the assigned intervention strategy, we hoped to increase adherence especially to the reappraisal strategy, as the results of Study 1 indicated that perhaps students did not effectively remember this strategy at the end of the intervention quarter. In Study 2, we administered similar interventions to students in a more diverse, suburban environment, to see if the results from Study 1 would generalize to other educational contexts.

## Supplementary Methods 2

*School 2 Participants.* Students were recruited from a suburban school in the Washington, D.C. area, and from approximately 272 students enrolled in Algebra I, Algebra II and Algebra II Honors classes, 167 students had parents/guardians who provided consent to participate in this study (59% response rate, 6 students not enrolled in study due ineligibility because they enrolled in a different math class). Five students did not have complete grade information, and were excluded from the dataset, for a total of 156 students who were included in the dataset for analysis ($N = 156$, 53% female; $n = 21$ Algebra I students, 43% female; $n = 100$ Algebra II students, 58% female; $n = 34$ Algebra II honors students, 44% female). This sample was considerably more racially diverse than Study 1, and was 52% white, 6% black/African American, 15% Hispanic/latino, 19% Asian, and 6% Native Hawaiian/Other Pacific Islander. Approximately 12% of the sample was economically disadvantaged (qualified for free or reduced school lunch). Overall, the school composition is 60% minority (40% white, 10% black/African American, 20% Hispanic/Latino, 24% Asian, 5% multiracial, and <1% Hawaiian Native/Pacific Islander/American Indian/Alaskan Native), 51% of students are female, and 25%

of students are economically disadvantaged (US News and World Report). Across the student body of the high school, 63% are rated as proficient in math, 22% are rated as failing, and 15% are advanced in mathematical proficiency based on results from the state-wide standardized testing (US News and World Report). Students were recruited from 13 classes taught by six instructors; all classes are year-long courses and no students was enrolled in more than one math class.

As in Study 1, all students were pseudo-randomly assigned to intervention technique. To account for effects created by class or teacher, each class was split in half, and half of the students assigned to each intervention condition. Similar to Study 1, assigned groups were counterbalanced by previous semester's grade performance and gender distribution so that they would be roughly equivalent within each class in gender composition and previous academic performance before the intervention was assigned (ER group: $n$ = 77, 52% female, average semester 1 grade: 81.47%, SD: 12.29% ; SS group: $n$ = 79, 54% female, average semester 1 grade: 81.44%, SD: 12.13%).

All study procedures were approved by the Committee for the Protection of Human Subjects and the review board associated with the local school district. All parents/guardians of students enrolled in the study provided a signed informed consent, and all students gave verbal assent to participate in the study. As all study procedures were performed as part of normal classroom procedures, students did not receive monetary compensation for this study in agreement with school district regulations.

*School 2 Design.* As with Study 1, the intervention strategies were introduced during the second semester of the school year, at the beginning of the third quarter (Supplementary Figure 1). Unlike Study 1, in this sample, all students were enrolled in year-long math courses

(compared to students in Study 1 who were enrolled in both semester-long and year-long courses). Class performance during the first semester was used as a baseline from which we could detect changes in grade performance (within-subject changes in performance) for each intervention group (between-subject comparison). As in Study 1, students were assigned to a specific intervention strategy, received in-class instruction on their assigned strategy in small groups at the beginning of the third quarter, and in this study we chose to increase the frequency of the reminders about the assigned intervention strategy throughout the intervention quarter. As a change from Study 1, in this sample we chose to add additional reminders about the assigned intervention in order to combat some of the adherence problems (forgetting the strategy) observed with the ER strategy in Study 1. In this sample, students were asked to fill out experience sampling questions and complete a short worksheet reminding them about their assigned intervention strategy during their study hall periods (approximately every 10 days) throughout the intervention quarter. Additionally, instead of only implementing the pre-test writing intervention during one test, students were also asked to use this strategy on all tests throughout the intervention quarter. In this way, we hoped that more frequent reminders about these strategies would lead to further increases in math class performance. In order to measure performance in this sample, we utilized classwork and homework grades, test grades, cumulative quarter grades, cumulative semester grades, and performance on a state standardized test in Algebra I or II, respectively. Although we did not include reminders about the intervention strategy throughout the fourth (final) quarter of the year, we continued to follow grade performance to examine longitudinal effects of the interventions.

*School 2 Intervention Strategies.* The intervention strategies utilized in this sample were largely similar to those introduced in Study 1, with each strategy focusing on implementing

reappraisal or effective study skills. However, in Study 2, we streamlined some of the instructions to focus specifically on explaining two techniques for using each intervention (see Appendix for in-class intervention worksheets). The SS intervention focused on two study skills: spaced studying (Carpenter et al., 2012) and retrieval practice (Agarwal, D'Antonio, Roediger, McDermott, & McDaniel, 2014b; Bjork & Bjork, 2011; Roediger & Karpicke, 2006a; Weinstein et al., 2010). In spaced studying, students were encouraged to avoid cramming, and set aside time to review key information on a regular basis. In retrieval practice, students were encouraged to practice bringing information to mind by using self-testing, such as doing practice problems, taking practice quizzes or tests, and making flashcards as effective ways to study for their math class. This SS intervention did not differ in content from Study 1, but focused more specifically on teaching these two techniques to students.

In the ER intervention, students were again instructed how to use cognitive reappraisal in academic settings, such as math class. As in Study 1, this reappraisal strategy focused on using a *distancing* strategy ("Imagine you're explaining the problem to your best friend"), but also introduced a *reframing* technique that aims to change the cognitive appraisals associated with a stress response, instead focusing on the possible positive associations with increased physiological arousal ("Think about the situation as a challenge rather than an obstacle," "Stress may help you perform better, use these feelings to help you focus and overcome this challenge") as has been used in previous research linking reappraisal to better academic performance under stress (Jamieson et al., 2010; 2012; 2016; Jamieson, Mendes, & Nock, 2013). Please see Appendix for in-class handouts.

Again, when these intervention techniques were introduced, students were asked to describe ways that they would utilize these techniques, and any off-topic responses were

redirected by the experimenters. Both intervention techniques focused on presenting two strategies for implementing the assigned technique, and students received reminders about these techniques throughout the intervention quarter by completing worksheets during study hall periods approximately every 10 days (see Appendix for worksheets). In addition to completing short experience-sampling surveys during these study hall periods, students read a short description of their intervention technique, and described in writing how they would use this technique. Students were also asked to briefly write about how they would implement these techniques immediately before all the exams given during the intervention quarter (for further description of this writing task, see "Midterm Intervention" in Study 1). Students completed the same set of surveys utilized in Study 1 (see Study 1 "Surveys"). Changes made to the structure and content of the intervention for Study 2 were meant to maintain similar content to Study 1, but to streamline the instructions so that they would be better understood by the students, and further reinforce these techniques over the course of the intervention quarter, so that the techniques would be better remembered and more likely to be implemented by the students.

## Supplementary Discussion 2

All analyses were completed with R statistics (Team, 2008). Linear mixed models were utilized to examine effects of the interventions as well as individual differences in the students using package lme4 (Bates et al., 2018) and lmerTest (Kuznetsova, Brockhoff, & Christensen, 2017). Reported fixed effects in linear mixed models (LMMs) are reported using Type III Wald $\chi^2$ tests to estimate the statistical significance and interactions between these fixed effects.

*School 2 Grades.* In this analysis, we focused on math class grades that reflected homework and classwork performance (including homework, quizzes, but not tests), as these grades reflected only grade performance during the intervention quarter; cumulative quarter

grades also included previous course performance from the first semester. Here we used linear mixed models to compare grade performance over the course of the intervention quarter, and the subsequent final quarter. This analysis allowed us to examine how the different intervention techniques influenced grade performance while the intervention was being implemented, as well as examining whether these effects persisted after students were no longer receiving active reminders to utilize these intervention techniques. Because this sample was larger and included a greater variety of classroom environments, we also chose to control for a number of factors, such as class subject matter and instructor. Both class subject matter (comparing Algebra I, Algebra II, and Algebra II honors, main effect of subject, $\chi^2(2) = 29.53$, $p < .001$) and instructor (comparing 7 instructors, main effect of instructor, $\chi^2(4) = 42.24$, $p < .001$) had a significant influence on grade performance across second semester when these factors were included in linear mixed models (LMMs) as fixed factors (performance evaluated with participant entered as a random effect). Neither of these factors interacted with the intervention group, but were included as random effects in the models comparing grade performance to account for these significant differences in grade performance. We also included a measure of previous performance (previous quarter classwork grades) to control for baseline differences in academic performance before the interventions were introduced in models comparing performance across the second semester.

To evaluate the effects of the intervention on grades, we used a linear mixed model with intervention group (2: SS intervention, ER intervention) and quarter (2: intervention, final) as fixed effects, and random effects accounting for each individual, the subject matter of the class, the instructor, and previous class performance (previous quarter classwork grade). This is a more conservative replication of the longitudinal analysis used in Study 1, because in addition to

accounting for previous performance, we also accounted for differences across classes, instructors, and previous levels of grade performance that could have influenced the effectiveness of the intervention. In this LMM (REML criterion: 2298.1), we find a main effect of quarter (fixed effect) on grade performance, such that overall, grades were higher during the intervention quarter ($M = 80.65$, $SE = 7.18$) than the final quarter ($M = 75.14$, $SE = 7.18$), $\chi^2(1) = 19.90$, $p < .001$. There was no main effect of intervention group, $\chi^2(1) = .84$, $p = .35$, and no interaction between quarter and intervention group, $\chi^2(1) = .69$, $p = .40$. Overall, we find that grade performance is elevated during the intervention quarter, though this did not differ by intervention group.

This analysis did not replicate the previous pattern observed in Study 1 in which the SS intervention group showed group-wise increases in performance during the intervention quarter compared to the final quarter. When we look at the performance of the groups across the school year (lmm with group and all 4 quarters entered as fixed effects, with random effects for each individual, the course subject matter and instructor), again we find an effect of quarter on grades, $\chi^2(3) = 63.65$, $p < .001$, with no main effect of intervention group, $\chi^2(1) = .54$, $p = .46$, and no interaction of quarter and intervention group, $\chi^2(3) = 3.07$, $p = .38$. As in Study 1, overall, grades have a downward trend across the school year ($M_{Q1} = 85.86$, $SE_{Q1} = 4.57$; $M_{Q2} = 82.50$, $SE_{Q2} = 4.57$; $M_{Q3} = 80.20$, $SE_{Q3} = 4.58$; $M_{Q4} = 74.76$, $SE_{Q4} = 4.58$). While it is possible that both interventions may have resulted in a boost for grades compared to the final quarter, it appears as though these temporal changes in grades are part of the downward slope of grades over the course of the year.

*School 2 Demographic Factors.* As in Study 1, we compared performance across gender in these classes to evaluate whether female students may have experienced stereotype threat in

math classes, leading to underperformance in grades. To evaluate grade performance, we computed a LMM with gender, group, and quarter as fixed factors, and random effects associated with each participant, subject matter, instructor, and previous grade performance. In this model we do find a main effect of gender, $\chi^2(1) = 5.74$, $p = .02$, such that female students performed better ($M = 82.56$, $SE = 7.47$) than the male students overall ($M = 74.20$, $SE = 7.40$). As discussed in other similar models (see above), there way no main effect of group, $\chi^2(1) = .99$, $p = .31$, and a main effect of quarter on grades, $\chi^2(1) = 19.58$, $p < .001$. There was a significant interaction between gender and quarter, $\chi^2(1) = 4.53$, $p = .03$, such that although both groups declined in grade performance from the intervention quarter ($M_{males} = 78.30$, $\mathrm{SE}_{\mathrm{males}} = 7.46$; $M_{females} = 84.00$, $SE_{females} = 7.52$) to the final quarter ($M_{males} = 70.08$, $\mathrm{SE}_{\mathrm{males}} = 7.46$; $M_{females} = 81.12$, $SE_{females} = 7.52$), this drop was more exaggerated for males, and females always had better grade performance than the males enrolled in the class. All other interactions (gender x intervention group, intervention group x quarter, and the three way interaction, gender x intervention group x quarter) were not significant, all *p*'s > .3. Female students did report slightly higher math anxiety than males (AAI-Math scores, $M_{females} = 2.98$, $M_{males} = 2.72$), though this increase is not statistically significant, $t(124.94) = 1.80$, $p = .07$[5]. These results replicate what was found in Study 1, and we can determine that if female students are experiencing any stereotype threat, it does not cause these female students to underperform relative to their male counterparts.

*School 2 Individual Differences in Anxiety.* In Study 2, we again sought to understand how the two intervention strategies would interact with students' anxiety levels to influence their

[5] We hypothesize it is the case that this increased math anxiety is a result of females self-reporting higher levels of anxiety in general in this sample. Female students report significantly higher trait anxiety, $t(127.76) = 3.11$, $p = .002$, and test anxiety, $t(123.62) = 4.63$, $p < .001$.

grade performance. In Study 2, due to the larger size of the dataset, and longitudinal nature of the classes (all classes were year-long), we utilized analyses that would allow us to explore performance differences across the second semester after the interventions were introduced, while controlling for performance during the first semester (before the interventions). One of the strengths of Study 2 is the ability to examine longitudinal effects of the intervention using the first semester, which occurred before the interventions were introduced, as a baseline. To this end, we additional compared the effects of anxiety on performance comparing semester one (pre-intervention) to semester two (intervention).

Using a LMM with anxiety, group and semester as fixed factors, and random effects for individual participant, subject matter and instructor, we evaluated how anxiety level and group would influence grade performance. We first evaluated this model with test anxiety (AAI-Test), and we find a main effect of term, $\chi^2(1) = 5.62$, $p = .02$, though this is subsumed by an AAI-Test by term interaction, $\chi^2(1) = 6.69$, $p = .01$, all other main effects (group, term, and AAI-Test) and interactions were not significant, all p's > .2. During the pre-intervention term, grade performance is negatively associated with increased test anxiety (Supplementary Figure 6), and during the intervention term, this relationship is diminished, such that anxiety no longer predicts decreased performance. This result suggests that the introduction of both intervention techniques reduced the negative impact of anxiety on grade performance during the second semester. For other measures of anxiety, such as test anxiety and trait anxiety, we do not find the same longitudinal interaction with term and anxiety, *p*'s > .3.

To further understand how these effects influence performance during the course of the intervention, we examined performance during second semester, comparing the intervention quarter to the final quarter, while controlling for previous performance. We used LMMs to

evaluate how each intervention would interact with anxiety over the intervention quarter and final quarter in the second semester of the math class. Each LMM included the intervention group, individual differences in anxiety, and quarter as fixed effects, and random effects for each participant, the class subject matter, instructor, and previous class performance (during second quarter).

First we evaluated the relationship between intervention group, quarter, and math anxiety, measured by the AAI-Math scale (fixed factors, random effects for each participant, subject matter, instructor, and previous class performance). In this model, we find a trending main effect of AAI-Math on grade performance, $\chi^2(1) = 2.99$, $p = .08$, such that as MA increases, grades decrease. We find no significant effect of quarter on grades in this model, $p > .3$. We find a significant effect of intervention group on grades overall, $\chi^2(1) = 5.88$, $p = .01$, such that overall, the ER intervention group had higher grades during second semester ($M = 77.30$, $SE = 7.68$) than the SS intervention group ($M = 74.47$, $SE = 7.59$). There is no significant interaction between AAI-Math and quarter, and no significant interaction between interaction group and quarter, all $p$'s $> .3$. We also find a significant interaction between AAI-Math scores and intervention group, $\chi^2(1) = 5.11$, $p = .02$, such that across grades for both the intervention quarter and final quarter there is a negative relationship between AAI-Math scores and the ER intervention group (this relationship does not change across quarters, so the three way interaction is not significant, $p > .3$). In this interaction between MA and intervention group, increased anxiety is associated with performance deficits for those in the ER group who are high in MA. However, for the SS group, this negative relationship is diminished, so that we no longer observe the same performance deficits for students who are high in MA. This interaction mirrors what was found in Study 1 with both trait anxiety and test anxiety, that the SS intervention reverses performance deficits

associated with increased anxiety, bolstering performance for students how are high in anxiety.

In other to further unpack this effect, we also examined this interaction only during the intervention quarter using a linear model (no additional random effects), we find a trending main effect of intervention group, $F(1,114) = 3.67$, $p = .06$, such that grades in the ER intervention group were slightly higher ($M = 78.10$, SE = 2.59), than those in the SS intervention group ($M = 76.62$, $SE = 2.52$). We also find a significant main effect of AAI-Math scores on grades, $F(1,114) = 7.64$, $p = .007$, such that higher MA scores are associated with decreased performance. We also find a trending interaction between intervention group and AAI-Math scores for classwork grade performance, $F(1,114) = 3.31$, $p = .07$ (Supplementary Figure 7). As in Study 1, here we observe that for the ER intervention group, although mean performance of this group is overall slightly higher, we still observe that as anxiety increases, grade performance decreases. However, for the SS intervention group, as in Study 1, again we observe that the SS intervention reduces the impact of anxiety on grades, such that those students who are higher in anxiety no longer show deficits in performance.

In replicating Study 1, we examined other effects of anxiety on performance, we computed a similar LMM with fixed effects for interaction group, test anxiety (TAI), and quarter (random effects for individual participant, subject matter, instructor, and previous grade performance). In this case, we only find a significant effect of quarter on grades, $\chi^2(1) = 5.78$, $p = .02$, such that across both groups and all levels of anxiety grades were higher during the in the intervention quarter ($M = 79.58$, $SE = 7.68$) than the final quarter, ($M = 73.87$, $SE = 7.68$). All other fixed effects (TAI scores, intervention group), and interactions (TAI x quarter, TAI x intervention group, quarter x intervention group, and TAI x quarter x intervention group) were not significant, all $p$'s > .2. We also explored effects of test anxiety using the AAI-Test subscale

and found similar results, suggesting a trending effect of quarter on classwork grades, $\chi^2(1) = 3.69$, $p = .06$, and no other significant effects or interactions of fixed effects, all $p$'s > .1. The results relating to test anxiety found in Study 1 did not replicate in Study 2, and instead are more similar to the interaction shown in math anxiety in Study 2.

Similar to the LMM exploring test anxiety, we explored trait anxiety in a LMM with fixed effects for interaction group, test anxiety (STAI—trait subscale), and quarter (random effects for individual participant, subject matter, instructor, and previous grade performance). In this model, there were no significant main effects of any fixed effect, and no interactions, all $p$'s > .1. We also explored effects of trait anxiety using the AAI-Trait subscale and found similar results, suggesting a trending effect of quarter on classwork grades, $\chi^2(1) = 3.41$, $p = .07$, and no other significant effects or interactions of fixed effects, all $p$'s > .1. As with test anxiety, the interaction between group and trait anxiety found for grades in Study 1 is more similar to the results in Study 2 related to math anxiety.

We also examined how other measures of anxiety might interact with group over the quarters, but found no significant effects of any fixed factor in models that included the MARS, AAI-Science or AAI-Writing as fixed effects, all $p$'s > .08.

*School 2 Emotion Regulation Strategies.* As in Study 1, we used scores on the reappraisal and suppression subscales of the ERQ (Gross & John, 2003) to compare habitual emotion regulation strategies across both intervention groups. Examining these effects only during the intervention quarter, a separate ANOVA for each subscale was calculated to examine the effects of intervention group and reappraisal or suppression on intervention quarter grades. There was no significant main effects or interactions (ERQ-ES, intervention group, or ERQ-ES x intervention group) for the suppression subscale, all $p$'s > .3. There was no significant main

effects or interactions (ERQ-CR, intervention group, or ERQ-CR x intervention group) for the reappraisal subscale, all $p$'s > .2. Using LMMs to examine whether suppression and/or reappraisal would influence performance across the second semester after the intervention techniques were introduced. In a LMM for suppression (ERQ-ES, intervention group, and quarter entered as fixed effects, subject matter, instructor, and previous grade performance as random effects), we found no main effects associated with suppression, and no interactions, all $p$'s > .2 (the only significant effect was associated with quarter). In a LMM for reappraisal (ERQ-CR, intervention group, and quarter entered as fixed effects, subject matter, instructor, and previous grade performance as random effects), we found no significant effects associated with any of the fixed factors, all $p$'s > .2. Overall, these results indicate that we were not able to replicate our finding in Study 1 related to positive effects of habitual emotion regulation in the SS intervention group.

*School 2 Adherence: Writing Samples.* One of the improvements we made in Study 2 compared to Study 1 was increasing the frequency with which students received reminders about their intervention, with the hope that students would show improved adherence to the strategies, especially in the ER intervention. Since the results of Study 1 indicated that students in the ER intervention had difficulty remembering the reappraisal strategy later in the term, students completed worksheets to remind them what was involved with their strategy during study hall sessions, as well as completing similar short surveys and writing samples during these study hall sessions and immediately before testing sessions. Despite being given instructions for when teachers were to administer these short surveys and worksheets, it was unclear exactly when these surveys and writing samples were administered, due to snow days, absences, and teacher administration error. As such, due to some ambiguity about what points during the term the

writing/reminder tasks were administered, we will not be formally analyzing the content of the writing samples for Study 2 for content.

Anecdotally, students in the ER intervention were better-able to produce responses in the writing samples that were coherent with the reappraisal strategy they had been taught in Study 2, writing responses such as, “Think about the problem like a game you want to beat,” “Pretending this is a preassessment, and prepare for success,” “Distancing - take the problem step-by-step. Think out what I'm doing + why (like I'm explaining it to my friend); Reframing - take my stress and use it as motivation to do,” “Distancing: Try to remember [instructor] explaining and going through the problem. Reframing: Think of each problem as a challenge,” “Imagine that the teacher is teaching me to do the problem; Imagine your doing the problem with a friend.” That any students were able to remember and use these ER strategies throughout the term shows a significant improvement over the writing responses provided in Study 1, almost none of which reflected knowledge of reappraisal. However, what is most important with regards to these reminders and writing samples is that they occurred with greater frequency throughout the term, increasing the likelihood that each student would remember and use their strategy, the efficacy of which was further evaluated by examining survey responses given during these reminders.

*School 2 Adherence: Experience Sampling Surveys.* In order to assess whether students opinions and experience might change throughout the course of the intervention, students completed surveys asking about their confidence associated with math, their understanding of the material, their math anxiety, and how frequently they used some of the techniques highlighted in the interventions: using a study schedule, using reappraisal to deal with stress, and using self-testing to study. Students completed these surveys during study hall periods as well as before tests given in class, and scores were averaged across periods of approximately ten days during

the intervention quarter. The first time point occurred when the intervention was introduced, the final time point was at the end of the intervention quarter. As mentioned previously, there was some ambiguity regarding the correct administration of these surveys, due to absences, snow days, and administration error, and as such, some participants did not answer surveys for all timepoints (e.g., only $n = 49$ participants provided responses during time point 4). Although our analysis methods (LMM) are relatively robust to account for missing data, that these data only represent a subsample of the participants tested in the study should be kept in mind as a limitation of these results.

Examining the interaction over time with LMMs, using intervention group and assessment time point as fixed factors, and random effects for individual participant, subject matter, and instructor, we evaluated how these opinions and behaviors changed over the course of the intervention quarter. We found no significant main effects or interactions for ratings of confidence, understanding, or anxiety, all $p$'s > .1. In a LMM examining reappraisal frequency ("Tried to rethink my perspective or change my feelings if I felt stressed," see Appendix), we found no significant effect of survey time point, $\chi^2(4) = 1.68$, $p = .79$, a trending effect of intervention group, $\chi^2(1) = 3.17$, $p = .07$, and a time point by intervention group interaction, $\chi^2(4) = 10.87$, $p = .03$. Although both groups report equivalent levels of reappraisal at the start of the intervention, the ER intervention group reports increased frequency of reappraisal over time compared to the SS intervention group (Supplementary Figure 8). When we evaluate two of the behaviors associated with the SS intervention group, scheduling and self-testing, we do not find a similar interaction with time point and intervention group, all p's > .3. Although we did not find increased adherence for the SS group for self-testing, as we did in Study 1, the increased frequency of reappraisal in the ER condition suggests increasing the number of reminders for the

intervention was an effective technique to encourage students to effectively encode the reappraisal intervention.

In Study 2, we again investigated the impact of introducing interventions focused on study skills or reappraisal strategies into high school math classrooms, specifically targeting performance deficits attributed to increased anxiety. In Study 2, our results again indicate that the SS intervention is advantageous for grade performance for highly anxious individuals. In examining the relationship between math anxiety, classwork grades, and each intervention, our results show that for the ER intervention, we observe a negative relationship between increased math anxiety and performance. However, for the SS intervention group, we find that the negative relationship between increased anxiety and grades is attenuated, such that increased math anxiety is no longer associated with performance deficits for students assigned to the SS intervention. However, although we observe a negative relationship between anxiety and performance for the ER group, when we compare the relationship between anxiety and performance before both interventions were introduced versus the semester when interventions were introduced, we find a negative relationship between test anxiety and performance in the pre-intervention term, but that the strength of this relationship is reduced during the intervention term for both intervention groups. In other words, both interventions have a positive influence in reducing the negative impact of anxiety on performance, with the greatest effect in reducing performance deficits associated with anxiety seen in the SS intervention.

Study 2 provided a valuable extension of the results found in Study 1, indicating that the SS intervention was associated with increases in performance for anxious individuals. In Study 1, most of the increases associated with the SS intervention were associated with individuals who reported high test anxiety or high trait (general) anxiety. In Study 2, though we see increases in

performance associated with test anxiety when we compare performance from semester 1 (pre-intervention) to semester 2 (intervention), when we examine performance within the intervention term, it is with highly math anxious individuals that we observe the largest increases in performance. Although it is interesting and important that both samples show increases in performance in the SS intervention, it is unclear exactly why these deficit reductions are observed for trait and test anxiety in Study 1 and for math anxiety and test anxiety in Study 2. We conceptualize that anxiety in academic situations is multi-faceted, with different aspects of performance pressure, habitual negative experience with the subject matter, as well as other patterns of negative affect all resulting in different kinds of anxious experience associated with mathematics. Although we conceptualize math anxiety, test anxiety, and trait anxiety to have important conceptual and functional distinctions (Pizzie & Kraemer, 2015), these interventions were designed to flexibly address academic anxiety more broadly. In this way, it is possible that the interventions in Study 2 may have been more germane to math-related material because the reminders given to the students were more specifically targeted for math classes, whereas the interventions in Study 1, although they were also presented to math classes, may have been more generally effective at reducing negative affect in academic scenarios. Over both studies, the results indicate that these interventions specifically reduce anxiety-related deficits in math classrooms, affecting anxiety specifically related to mathematics as well as broader patterns of negative affect in academic anxiety.

Study 2 was also designed to address some of the limitations in Study 1. First, Study 2 increased the sample size from Study 1 by approximately 50%, and included a racially- and economically-diverse population of students in a different region of the country. That we still find similar results across differing populations of students bolsters the ecological validity of

these interventions. In Study 2, we also increased the frequency of the reminders, surveys, and writing prompts given to remind the students about their assigned intervention and to assess how the students were using the techniques in each intervention. Whereas in Study 1, students did not accurately remember how to use the reappraisal technique, the increased frequency of these interventions in Study 2 seemed to remedy this problem: students in the ER intervention reported increased frequency using the reappraisal technique to deal with stress, and review of the written responses indicated that students wrote responses that were consistent with the reappraisal technique that they had been taught, a significant improvement from Study 1.

Moreover, the increased frequency of these intervention technique reminders seems to have had important effects over the course of second semester, such that when we compare the relationship between test anxiety and grade performance during the semester before the intervention was introduced to the intervention semester, we find that performance deficits associated with increased anxiety were reduced overall in second semester. It is important to note that the administration of these reminders and writing interventions may have been inconsistent throughout the intervention, which presents a significant limitation in interpreting the results of the surveys or writing samples collected during these class periods. However, that the increased frequency of these reminders may have increased the effectiveness of the intervention to extend beyond the intervention quarter and throughout the second semester represents an important point if this kind of intervention is to be implemented in classrooms.

Overall, in Study 2, we observed similar results to those found in Study 1, illustrating that the study skill intervention is an effective strategy for students who are higher in anxiety to have better performance in their math classrooms. The increased frequency of the intervention reminders in Study 2 improved the efficacy of both of the interventions, encouraging students to

implement these techniques themselves and subsequently showed better performance.

## Supplementary Note 3

*Quadratic Models of Math Anxiety in Both Samples.* In these analyses, we compare the curvilinear (quadratic) effects of anxiety on class performance. Indeed, many studies do show a curvilinear or quadratic (or negative quadratic, to be specific) relationship between stress and anxiety and performance. To address this, we ran additional analyses with our data. Here, we used our linear mixed models (LMMs) to test the same main effects in the paper: 1) Evaluating the interaction between math anxiety and intervention group overall on grades, 2) Evaluating the interaction between math anxiety and group within each quarter. For each of these analyses, we fitted two polynomial functions, one fitting a linear model to AAI-math scores, and one fitting a quadratic (curvilinear) model to AAI-math scores. Then we compared the linear and quadratic model to one another using the anova() function in R, which allowed us to compare whether the quadratic model accounted for additional variance above and beyond the linear model. For the interaction between math anxiety and intervention group on grades, the quadratic model of math anxiety did not account for additional variance above and beyond the linear model of math anxiety, $\boldsymbol{X}^2$ (2) = 1.08, $p$ = .58. For the interaction between math anxiety, intervention group, and quarter on grades, the quadratic model of math anxiety did not account of additional variance above and beyond the linear model of math anxiety, $\boldsymbol{X}^2$ (4) = 4.80, $p$ = .31.

Supplementary Table 1 . Mean grade performance for each group for each instructor across the year.

| **Instructor/Intervention Group** | | **PRE-INTERVENTION QUARTER (Q1)** | **PRE-INTERVENTION QUARTER (Q2)** | **INTERVENTION QUARTER (Q3)** | **FINAL QUARTER (Q4)** |
|---|---|---|---|---|---|
| Instructor 1 | Group 1 | 87.64 (SE = 2.80) | 82.82 (SE = 3.82) | 80.69 (SE = 3.32) | 80.42 (SE = 3.01) |
| Instructor 1 | Group 2 | 84.88 (SE = 2.80) | 80.23 (SE = 3.82) | 79.08 (SE = 3.38) | 74.76 (SE = 3.07) |
| Instructor 2 | Group 1 | 71.78 (SE = 2.72) | 76.56 (SE = 3.72) | 63.17 (SE = 3.52) | 62.96 (SE = 3.20) |
| Instructor 2 | Group 2 | 73.41 (SE = 2.80) | 83.65 (SE = 3.83) | 78.52 (SE = 3.69) | 70.91 (SE = 3.35) |

Note. Mean quarter grade performance and standard error for each instructor within each randomly assigned intervention group. Approximately 26 students do not have grades available for the first semester (Q1 and Q2), as they were enrolled in a semester-long math course during Q3 and Q4, and do not have year-long grades. Whereas the interactions between group and instructor are statistically significant in Q3 and Q4, the pattern across the means for each instructor remains similar both before and after the intervention is introduced, indicating that these differences between instructors are relatively constant across the year's grades.

**Supplementary Figures**

Supplementary Figure 1. Design of classroom intervention over the semester.

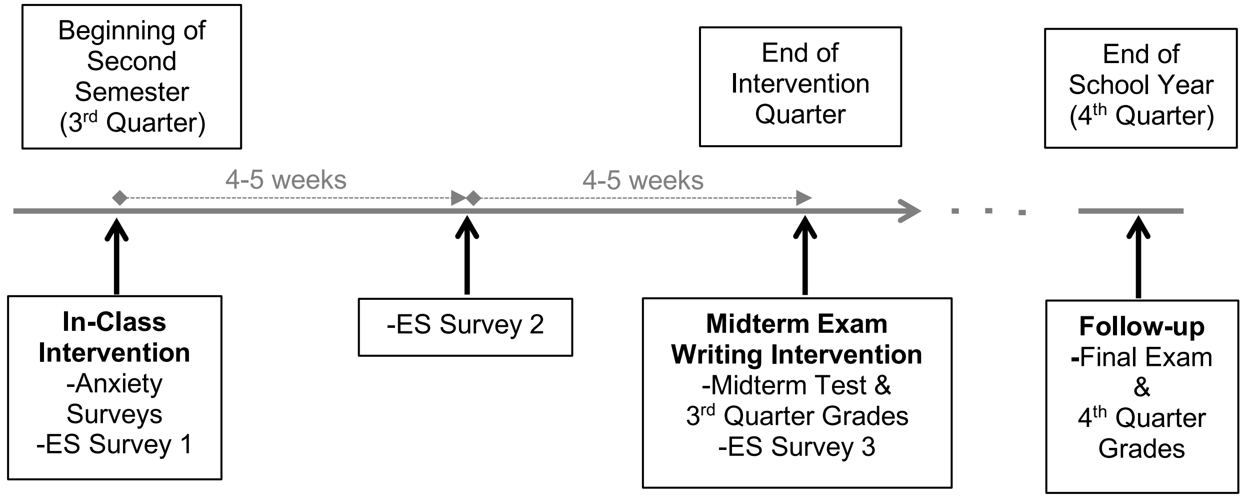


Note: Course of experimental procedures throughout the semester. Students first completed the in-class intervention in small group discussions and completed surveys. Experience Sampling (ES) surveys were given multiple times throughout the intervention (3rd) quarter. The midterm exam included the short expressive writing task and the final ES survey. Students were not contacted after the midterm, but researchers received final grades for the course.

Supplementary Figure 2. Grade performance for each intervention group during intervention and final quarters.

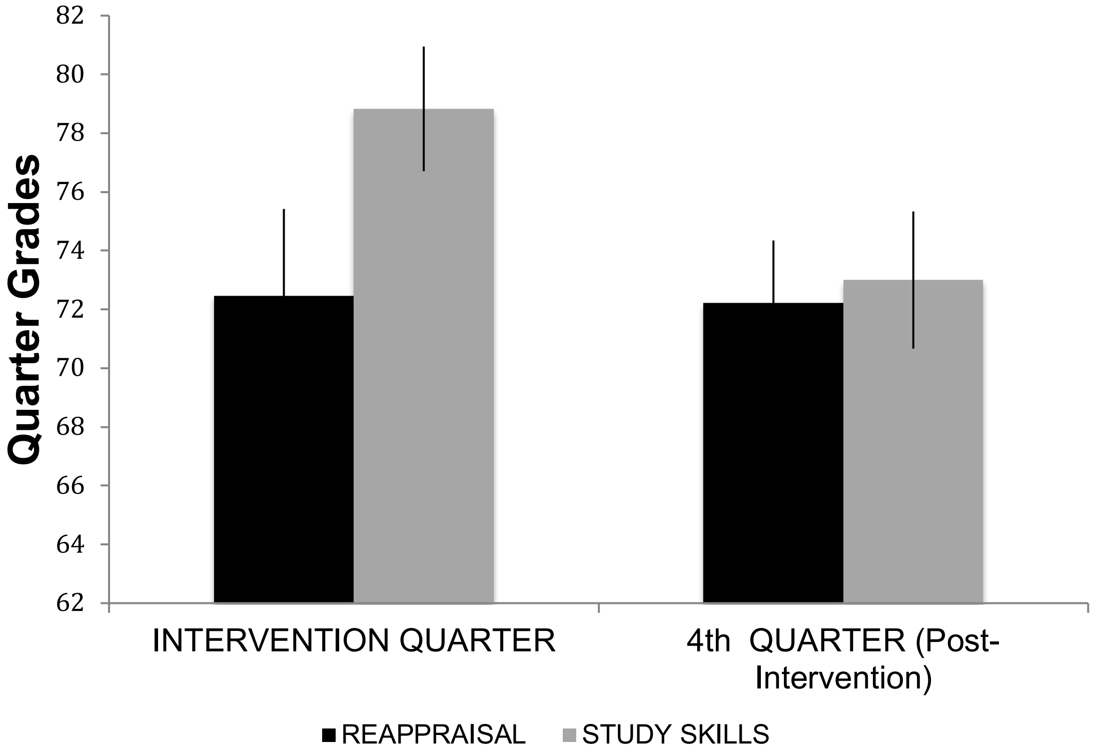


Note: Intervention quarter grades and final quarter grades for each intervention group. Grades in the SS intervention were improved during the intervention quarter, but were no different from the ER group for final quarter grades, $F(1,93) = 7.07$, $p = .009$, $\eta_p^2 = .071$. Error bars represent Standard Error.

Supplementary Figure 3. Interactions between intervention group and individual differences in anxiety for intervention quarter grade performance in Study 1.

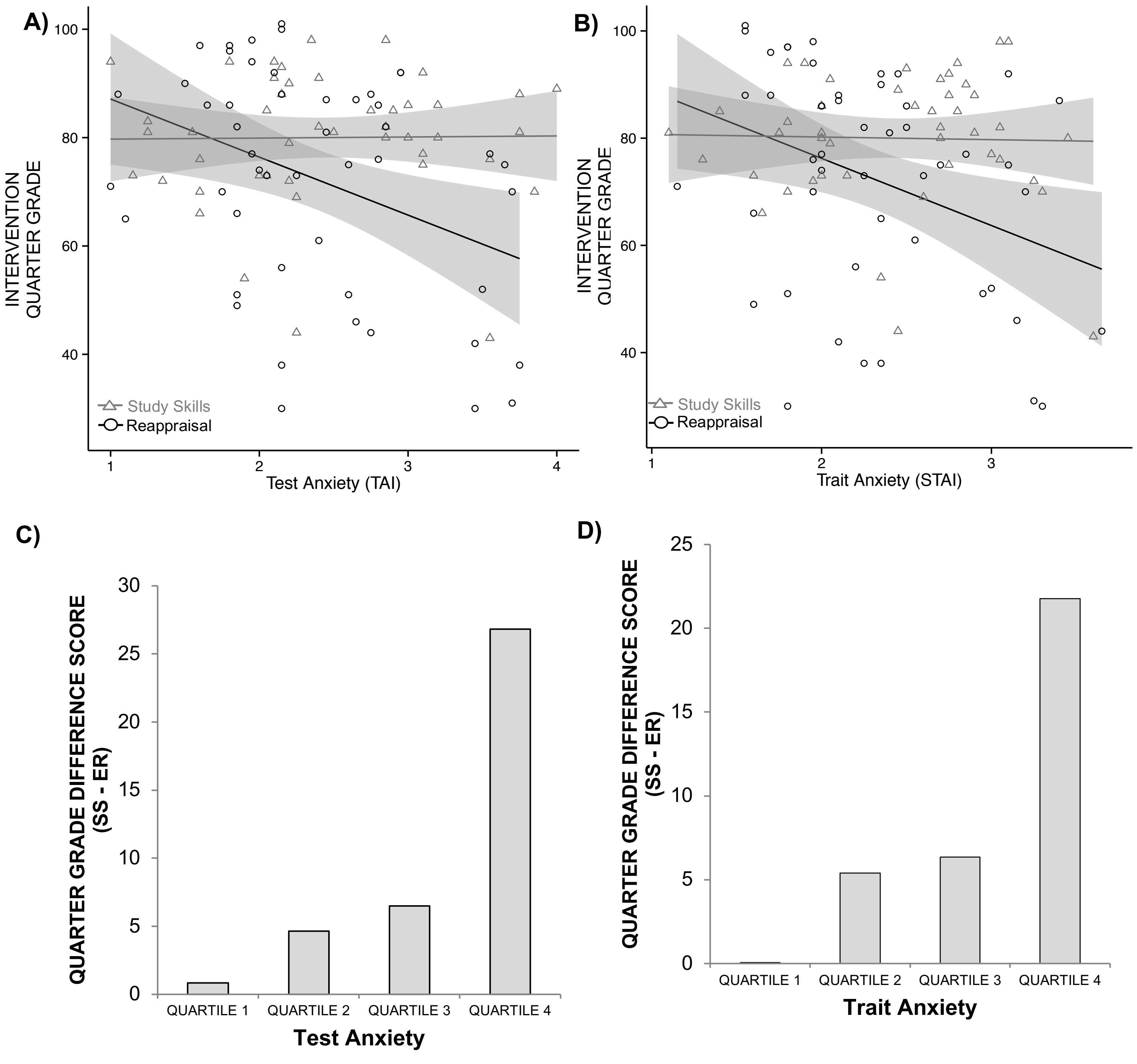


Note: Interactions between group and A) test anxiety, TAI: $F(1,90) = 5.69$, $p = .02$, and B) trait anxiety, STAI: $F(1,90) = 4.91$, $p = .03$.

Whereas students in the ER intervention show anxiety-related declines in performance, students in the SS intervention group do not show this decline in grades, even for students with high levels of anxiety. C) Increases in math performance in SS intervention compared to ER intervention for increasing levels of test anxiety illustrate the improvements for students high in test anxiety for intervention quarter grades (including homework, quizzes, and tests). D) Increases in the SS intervention compared to the ER intervention also illustrate the degree of improvement in intervention quarter grades, especially for those highest in trait anxiety. Error bars represent Standard Error.

Supplementary Figure 4. Interactions between intervention group and individual differences in emotion regulation on intervention quarter grades.

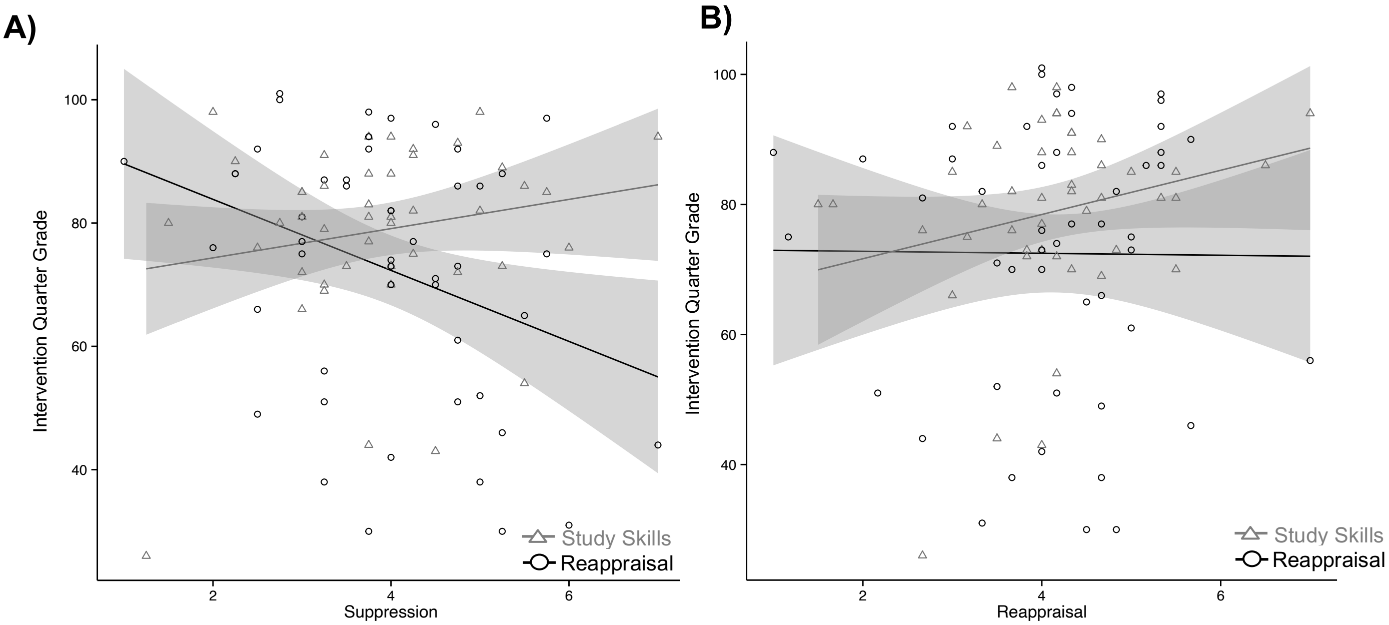


Note: Interactions between intervention group and self-reported emotion regulation tendencies. For both A) suppression, ERQ-ES: $F(1,91) = 7.02$, $p = .01$, and B) reappraisal, ERQ-CR: $F(1,91) = 1.06$, $p = .31$, the combination of more frequent use of emotion regulation strategies combined with the study skill intervention resulted in better performance. Error bars represent Standard Error.

Supplementary Figure 5. Interaction between intervention group and various timepoints during the intervention quarter for self-testing frequency.

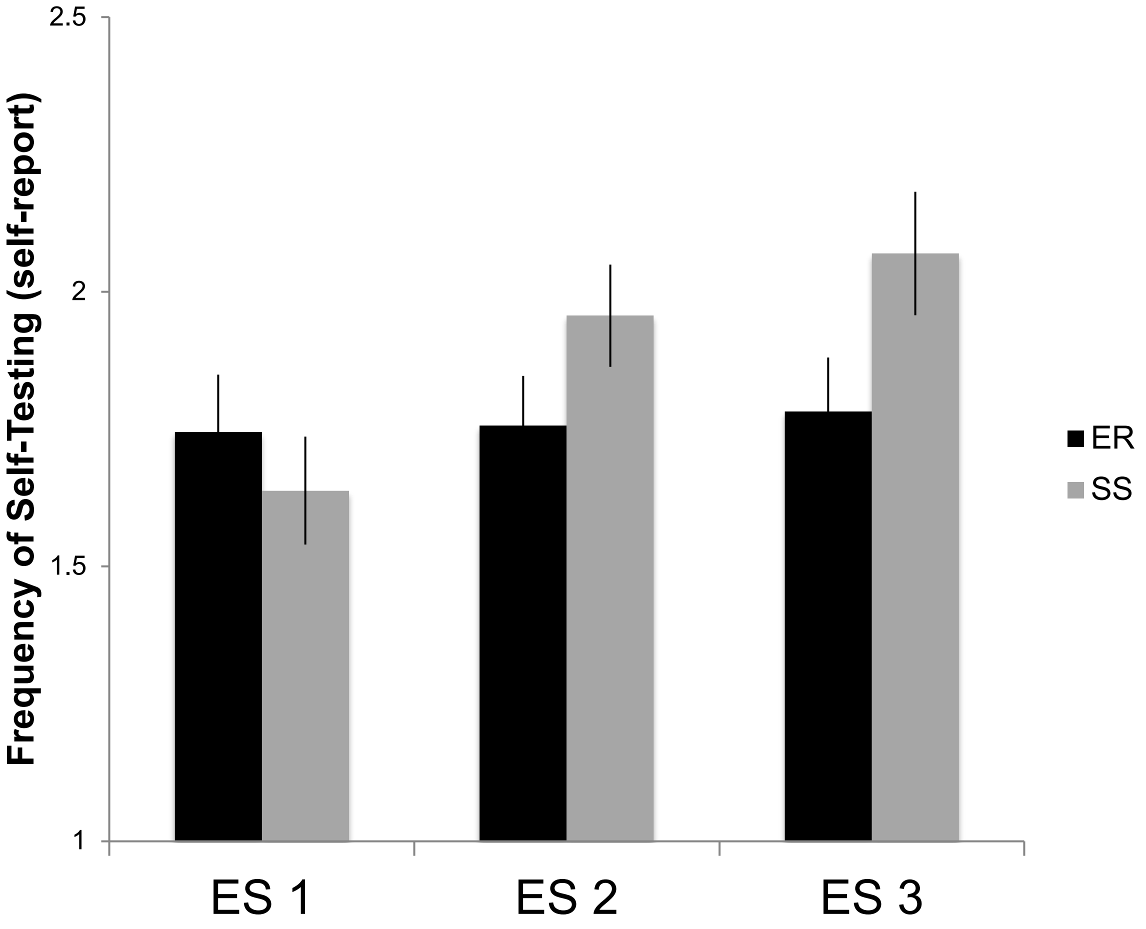


Note: Students in the SS intervention reported a significant increase in self-testing that was not observed in the ER intervention, $F(2,152) = 4.20$, $p = .017$, $\eta_p^2 = .052$. ES1 occurred at the beginning of intervention quarter, ES2 occurred approximately halfway through the intervention quarter, and ES3 data collection occurred immediately before the midterm exam, at the end of the intervention quarter. Error bars represent Standard Error.

Supplementary Figure 6. Interaction between semester and individual differences in anxiety for semester grade performance in Study 2.

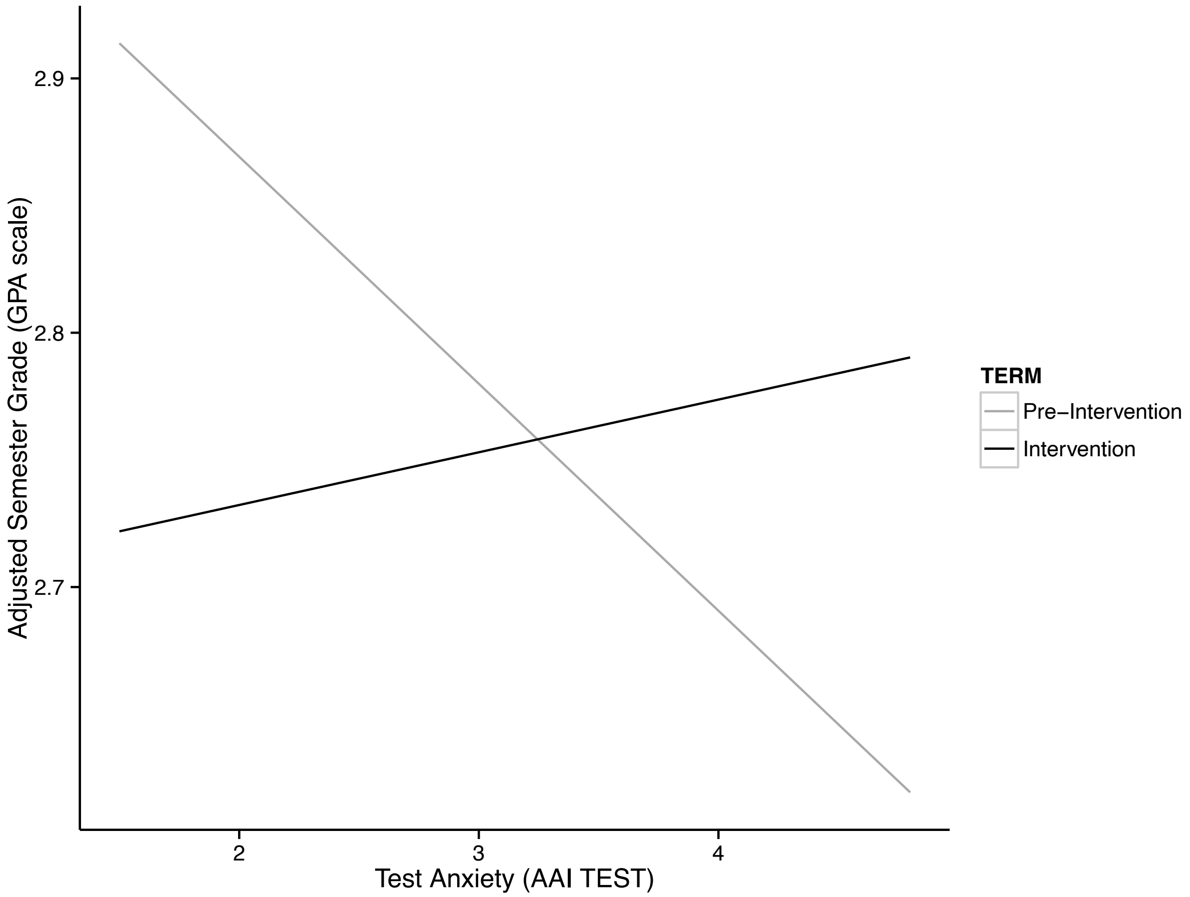


Note. Interaction between term and test anxiety (AAI-Test) on semester grades, $\chi^2(1) = 6.69$, $p = .01$. During the pre-intervention semester, we observe a negative relationship between increased test anxiety and performance, such that increased anxiety is associated with poorer grades. After the intervention is introduced, this negative relationship between anxiety and grades is ameliorated, so that highly anxious students no longer show a deficit in math performance. Importantly, this effect is across both intervention groups, so although we see more robust effects for the SS intervention in second semester, both interventions seem to have positive effects on anxiety and performance.

Supplementary Figure 7. Interactions between intervention group and individual differences in anxiety for intervention quarter grade performance in Study 2.

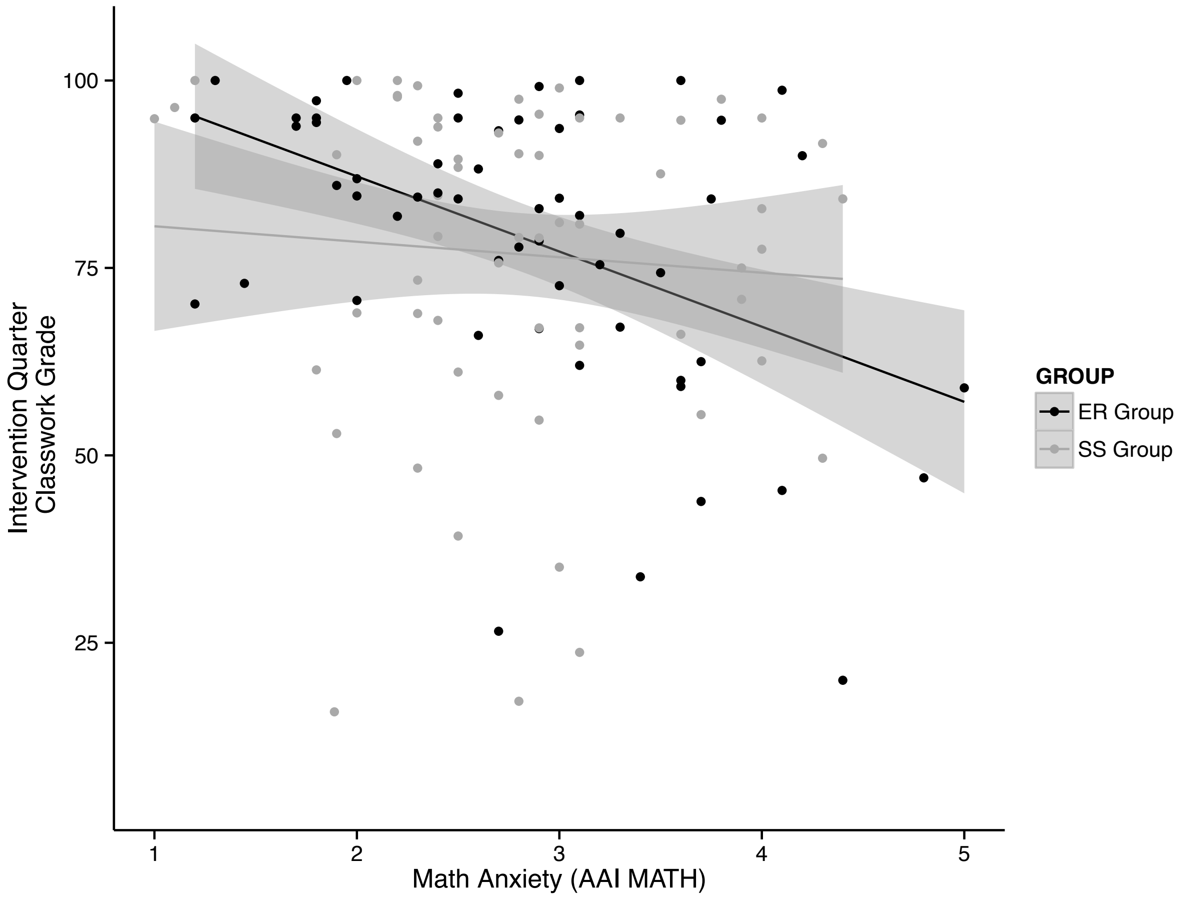


Note. Interaction between intervention group and math anxiety (AAI-Math), F(1,114) = 3.31, p = .07. For the SS intervention, anxiety has less of a negative impact on performance, such that highly math anxious individuals do not show performance deficits in mathematics (as observed in the ER group. This relationship persists through second semester, as evidenced by the interaction between AAI-Math scores and intervention group (LMM, fixed factors) for both quarters, $\chi^2(1) = 5.11$, $p = .02$. Error bars represent Standard Error.

Supplementary Figure 8. Interaction between intervention groups over time for self-reported frequency of reappraisal use.

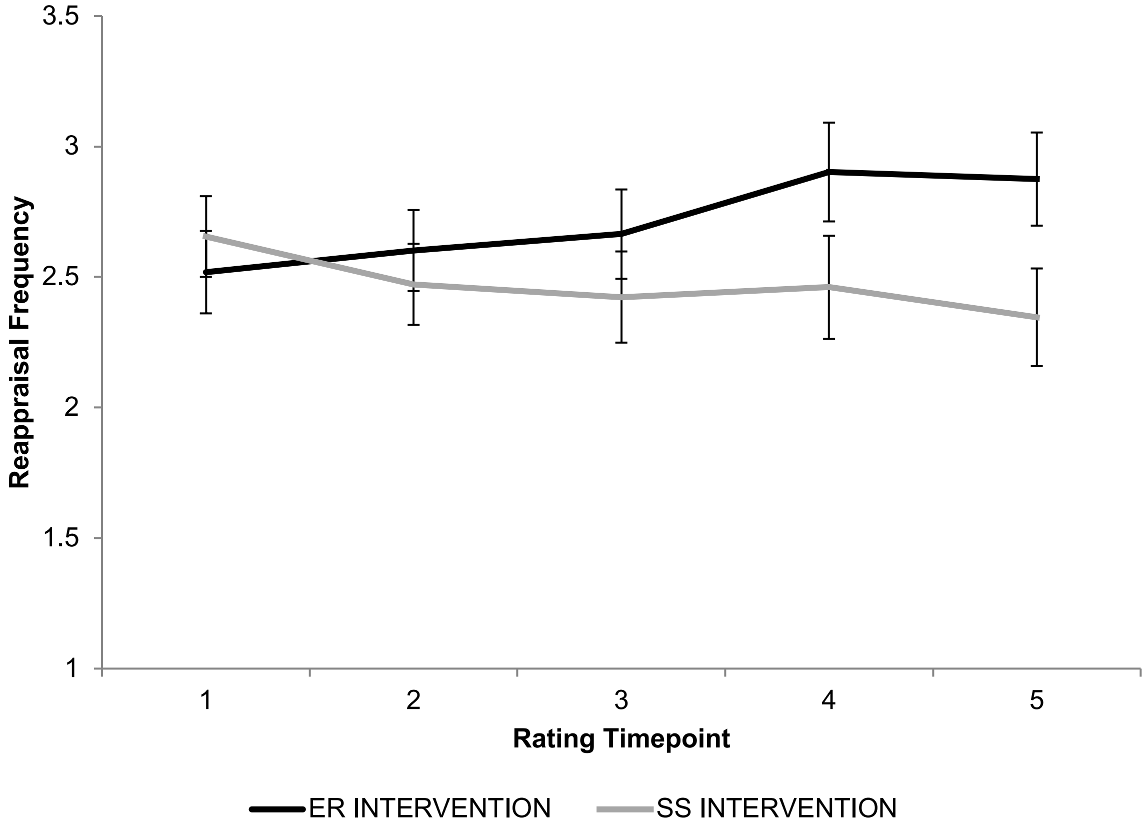


Note. Over the course of the intervention quarter (T1 occurs when the intervention was introduced, T5 occurs at the end of the intervention quarter) students in the ER intervention reported increased frequency of using reappraisal when faced with stress. Students in the SS intervention did not report increased frequency of reappraisal, and did not report increased frequency of self-testing, as reported in Study 1. Error bars represent Standard Error.

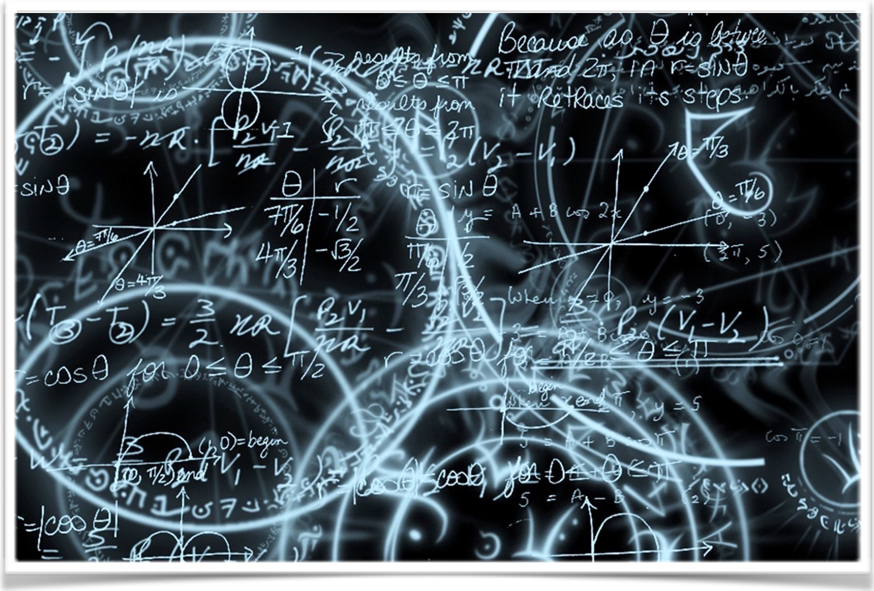

# Stress and Math

Focusing on dealing with stress and anxiety to improve performance in math class

## Responses to Stress

Can you think of a situation where you felt stressed out or anxious at school? What happened?

Why do you think it might be important to deal with your feelings of stress or anxiety?

# How can you effectively cope with stress?

**RETHINK YOUR EMOTIONS—***imagining a problem in a way that makes it less negative, or thinking about the situation from another perspective*

- **DISTANCING—**Think less about your own reactions or emotions in the situation, and more about the steps of the problem or the **bigger picture**
  - Focus on thinking **objectively**
  - If you use DISTANCING while working on math:
    - Instead of thinking about your stress or anxiety, **focus on solving each step of the problem as your teacher explained them to you**
    - Focus on someone else's perspective: **How would you explain this problem to a friend?**

**How can you use DISTANCING during math homework, quizzes and tests?**

## Thinking about the situation differently. How can you rethink your feelings about math?

- **REFRAMING—**change your perspective on your feelings and emotions
  - When you notice that you're feeling stressed, **think about your situation as a *challenge* rather than an *obstacle***
  - If you use REFRAMING while working on math:
    - Stress may help you perform better, **use these feelings to help you focus and overcome this challenge**
    - Feeling stressed can help you **solve problems more quickly and efficiently**

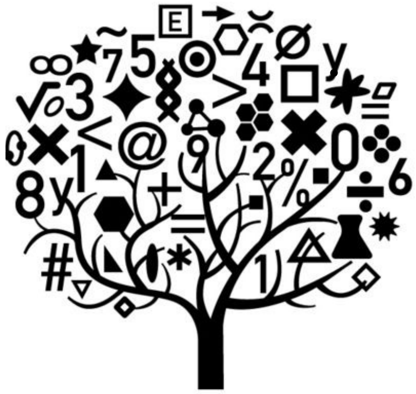

**How can you use REFRAMING during math homework, quizzes and tests?**

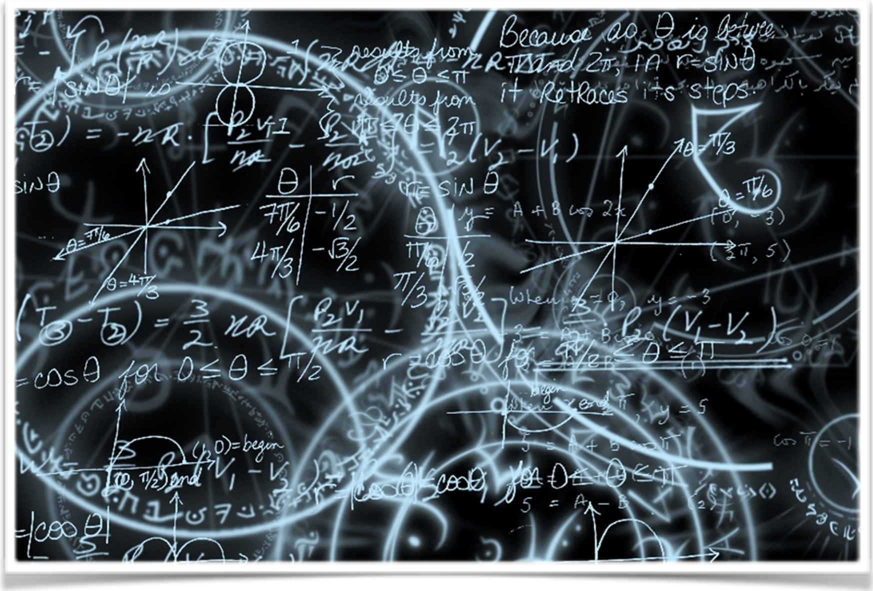

# Study Skills and Math

## Focusing on organization and study techniques to improve performance in math class

## Current Study Techniques

Can you think of a situation where you felt like you didn't have enough time or didn't know how to study? What happened?

Why do you think it might be important to improve how you manage your time, or improve the ways that you try to learn and review information?

# How can you make your studying more effective?

**Space out your learning using SPACED STUDYING!**

- **Spaced Studying**—Studying and reviewing the same topic over multiple days
  - **Avoid CRAMMING**: Plan for tests early, set aside a little time each day to study and review
  - Set aside time each day to **review information from each class**
  - After studying recent information, **go back and study key material from previous classes** to keep it fresh
- **Take breaks!**
  - Work for 45 minutes, take a 15 minute break

**How can you use SPACED STUDYING for math homework, quizzes and tests?**

## Plan and space out your study periods.

## Study by practicing—test your knowledge!

- **Test your knowledge by using RETRIEVAL PRACTICE—practice bringing information to mind**

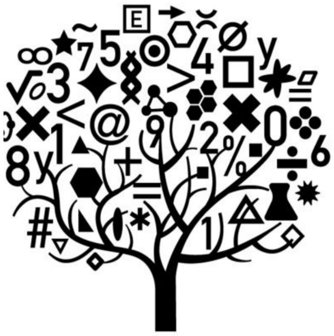

- **Practice DOING:**
  - Use practice problems in your book and online
  - Make flash cards and quiz yourself
  - Take practice quizzes or tests
- **Check your class materials for accuracy,** review things you may have missed

**How can you use RETRIEVAL PRACTICE for math homework, quizzes and tests?**

ID NUMBER:

# SURVEY

**DATE:** **CLASS:**

**For all of the following statements, please mark one option to indicate your response. Please answer all questions.**

**Please rate how much you agree with the following statements:**

I feel confident doing math.

| Strongly Disagree | Disagree | Neither Agree nor Disagree | Agree | Strongly Agree |
|---|---|---|---|---|

I don’t understand what I’m studying in math class.

| Strongly Disagree | Disagree | Neither Agree nor Disagree | Agree | Strongly Agree |
|---|---|---|---|---|

I feel anxious while I’m working on math.

| Strongly Disagree | Disagree | Neither Agree nor Disagree | Agree | Strongly Agree |
|---|---|---|---|---|

**In the past few days, please rate how often you've done the following activities:**

Tried to use a schedule to plan and organize my study habits

| Never | Once or Twice | Sometimes | Frequently |
|---|---|---|---|

Tried to rethink my perspective and change my feelings if I felt stressed or anxious

| Never | Once or Twice | Sometimes | Frequently |
|---|---|---|---|

Tried to review information by testing myself and practicing problems

| Never | Once or Twice | Sometimes | Frequently |
|---|---|---|---|

ID NUMBER:

Please think back on previous examples of different kind of emotions and thoughts that you have experienced in math class, and while studying on your own. In the space below, ***please write about some of your emotions, feelings and thoughts while working on math.***

**In the past week, how have you dealt with any feelings of stress?**

Earlier this semester, we talked with you about stress and negative emotions that can affect your performance in school, especially in math class. From the following options, what are some ways that you could use *EMOTION REGULATION* in order to change any feelings of stress you might have in math class?

a) Skip over problems I don't know and come back to them later.
b) Take a step back, remember I know how to do the problem, and think through the steps of the problem like I'm explaining it to my friend.
c) Think about the fact that some stress might be a good thing, and can help me perform better while I'm working on math.
d) Try to distract myself by taking a break, taking a few minutes to check my phone or email before coming back to try the problem again.

If you answered B or C, these are good emotion regulation techniques to reduce stress! Answer B refers to **DISTANCING,** or rethinking your perspective, for example, imagine you're explaining the problems a friend. Answer C refers to **REFRAMING YOUR FEELINGS,** for example, thinking about stress as a positive way to deal with challenges.
If you answered A or D, these are not effective emotion regulation techniques. Although these options are examples of a helpful test taking strategy (A), or might be you feel better by distracting yourself from stress (D), these strategies don't help you tackle the challenge you're facing right now.

***In your own words, please write***: *What are some ways that you can use DISTANCING or REFRAMING in order to rethink these feelings or change your perspective on these emotions while you're working on math?*

ID NUMBER:

# SURVEY

**DATE:** **CLASS:**

**For all of the following statements, please mark one option to indicate your response. Please answer all questions.**

**Please rate how much you agree with the following statements:**

I feel confident doing math.

| | | | | |
|---|---|---|---|---|
| Strongly Disagree | Disagree | Neither Agree nor Disagree | Agree | Strongly Agree |

I don’t understand what I’m studying in math class.

| | | | | |
|---|---|---|---|---|
| Strongly Disagree | Disagree | Neither Agree nor Disagree | Agree | Strongly Agree |

I feel anxious while I’m working on math.

| | | | | |
|---|---|---|---|---|
| Strongly Disagree | Disagree | Neither Agree nor Disagree | Agree | Strongly Agree |

**In the past few days, please rate how often you've done the following activities:**

Tried to use a schedule to plan and organize my study habits

| | | | |
|---|---|---|---|
| Never | Once or Twice | Sometimes | Frequently |

Tried to rethink my perspective and change my feelings if I felt stressed or anxious

| | | | |
|---|---|---|---|
| Never | Once or Twice | Sometimes | Frequently |

Tried to review information by testing myself and practicing problems

| | | | |
|---|---|---|---|
| Never | Once or Twice | Sometimes | Frequently |

ID NUMBER:

Please think back on previous examples of different types of math problems that you've seen in class, and the information that you've studied on your own. In the space below, ***please write down some of the kinds of problems you've been studying in math class:***

**In the past week, how have you studied for your math class?**

Earlier this semester, we talked with you about different study techniques you could use to improve your performance in math class. From the following options, what are some ways that you could use better STUDY SKILLS to change the ways that you study for math class?

a) Skip over problems I don't know and come back to them later.
b) Use a schedule to plan my study and review periods more often, going over new information every day and checking over old information.
c) Practice doing the problems using examples from the book, practice quizzes and practice tests.
d) Reread the chapter in the book, go over my notes from class, and use a highlighter to focus on the key information.

If you answered B or C, these are good study skills to use for math! Answer B refers to **SPACED STUDYING,** or reviewing a little bit at a time instead of cramming, for example, reviewing the material each day, and taking a short amount of time to review information from previous classes. Answer C refers to **RETRIEVAL PRACTICE,** for example, using practice problems and self-testing in order to learn and remember the information better over time.
If you answered A or D, these are not effective study strategies. Although these options are examples of a helpful test taking strategy (A), or might feel like you're reviewing the information (D), these strategies don't help you practice remembering and recalling the information you learned.

***In your own words, please write***: *What are some ways that* ***you*** *can use SPACED STUDYING or RETRIEVAL PRACTICE in order to learn, review and remember the material while you're working on math?*
