# Supplementary material for: Strategies for remediating the impact of math anxiety on high school math performance
Source: NPJ Sci Learn. 2023 Oct 2;8:44. doi: 10.1038/s41539-023-00188-5 (PMC10543627; doi:10.1038/s41539-023-00188-5)
Supplement: Supplementary file 2 — Reporting summary Checklist [file 41539_2023_188_MOESM2_ESM.pdf]

Corresponding author(s): Rachel Pizzie

Last updated by author(s): 2023-20-08

Reporting Summary

Nature Portfolio wishes to improve the reproducibility of the work that we publish. This form provides structure for consistency and transparency in reporting. For further information on Nature Portfolio policies, see our Editorial Policies and the Editorial Policy Checklist.

Statistics

For all statistical analyses, confirm that the following items are present in the figure legend, table legend, main text, or Methods section.

n/a	Confirmed	
☐	☒	The exact sample size (*n*) for each experimental group/condition, given as a discrete number and unit of measurement
☐	☒	A statement on whether measurements were taken from distinct samples or whether the same sample was measured repeatedly
☐	☒	The statistical test(s) used AND whether they are one- or two-sided<br>*Only common tests should be described solely by name; describe more complex techniques in the Methods section.*
☐	☒	A description of all covariates tested
☒	☐	A description of any assumptions or corrections, such as tests of normality and adjustment for multiple comparisons
☐	☒	A full description of the statistical parameters including central tendency (e.g. means) or other basic estimates (e.g. regression coefficient) AND variation (e.g. standard deviation) or associated estimates of uncertainty (e.g. confidence intervals)
☐	☒	For null hypothesis testing, the test statistic (e.g. *F*, *t*, *r*) with confidence intervals, effect sizes, degrees of freedom and *P* value noted<br>*Give P values as exact values whenever suitable.*
☒	☐	For Bayesian analysis, information on the choice of priors and Markov chain Monte Carlo settings
☐	☒	For hierarchical and complex designs, identification of the appropriate level for tests and full reporting of outcomes
☒	☐	Estimates of effect sizes (e.g. Cohen's *d*, Pearson's *r*), indicating how they were calculated

Our web collection on statistics for biologists contains articles on many of the points above.

Software and code

Policy information about availability of computer code

Data collection	For data collection, online forms and surveys were administered via Qualtrics online survey platform. For paper-and-pencil materials, responses were transcribed into spreadsheet software and summarized.
Data analysis	Data analyses were performed using Microsoft Excel and R statistical software.

For manuscripts utilizing custom algorithms or software that are central to the research but not yet described in published literature, software must be made available to editors and reviewers. We strongly encourage code deposition in a community repository (e.g. GitHub). See the Nature Portfolio guidelines for submitting code & software for further information.

Data

Policy information about availability of data

All manuscripts must include a data availability statement. This statement should provide the following information, where applicable:

- Accession codes, unique identifiers, or web links for publicly available datasets
- A description of any restrictions on data availability
- For clinical datasets or third party data, please ensure that the statement adheres to our policy

Data Availability. The materials associated with this project will be shared in the Open Science Framework online repository (https://osf.io/43q6y/). These data include educational data from underage minors. Sharing of these data is restricted based on FERPA regulations and research agreements with the participating local schools. Due to the nature of the data, deidentified data will be shared by request from the authors, but complete deidentified data will not be shared in an online

repository due to privacy restrictions and the sensitive nature of the educational data. Interested parties can contact the corresponding author, Rachel Pizzie (Rachel.pizzie@gallaudet.edu) to request access to the data and should receive a response to requests within two weeks. Interested parties will be asked to describe how they will ensure privacy and confidentiality of the data and maintain the security of the data. Interested parties must agree not to share these data beyond the parties included in the request, and do not have permission to publish the data themselves.

Research involving human participants, their data, or biological material

Policy information about studies with human participants or human data. See also policy information about sex, gender (identity/presentation), and sexual orientation and race, ethnicity and racism.

Reporting on sex and gender	Demographic information was provided by each school. For our analyses, we use the term "gender" to refer to masculine or feminine identity, as this encompasses the cultural and social context of gender roles, and our study does not refer to any biological measures of sex characteristics. The researchers realize there are a variety of identities that can be encompassed by gender identity, but for the purposes of these analyses, the researchers use the binary terms "male" and "female" to refer to the participants' gender identity as this was the information provided by each school. For additional information about this sample, please see the Supplementary Material.<br>Based on past research on gender and math anxiety, we conducted some analyses using gender as a factor of interest, but did not find any differences in our results attributed to gender.
Reporting on race, ethnicity, or other socially relevant groupings	Demographic information such as race/ethnicity was provided by the school. We did not analyze this factor as a part of our results. Instead of reporting individualized results, which have the potential to cross-identify participant information, we reported the school-level demographics. For school 1, Students were between the ages of 13 and 18 (Mage = 15.34, SDage = 1.05), and the sample was 60% female. Further demographic data such as race and measures of SES were not provided for this specific sample, but based on data from the 2014-2015 school year, the school is 99% white, 1% black, 1% Hispanic, and 41% of students are rated as economically disadvantaged (37% free lunch, 4% reduced-price lunch), and have 26% of students who are rated as proficient in mathematics (US News and World Report). In school 2, this sample was considerably more racially diverse than Study 1, and was 52% white, 6% black/African American, 15% Hispanic/latino, 19% Asian, and 6% Native Hawaiian/Other Pacific Islander. Approximately 12% of the sample was economically disadvantaged (qualified for free or reduced school lunch). Overall, the school composition is 60% minority (40% white, 10% black/African American, 20% Hispanic/Latino, 24% Asian, 5% multiracial, and <1% Hawaiian Native/Pacific Islander/American Indian/Alaskan Native), 51% of students are female, and 25% of students are economically disadvantaged (US News and World Report). Across the student body of the high school, 63% are rated as proficient in math, 22% are rated as failing, and 15% are advanced in mathematical proficiency based on results from the state-wide standardized testing (US News and World Report). Please see Supplementary Material for more information.
Population characteristics	See "Research Sample" section.
Recruitment	Participants were recruited from two geographically and demographically dissimilar public high schools within the United States. Participants were recruited in collaboration with the local school administration for both School 1 and School 2. In both districts, parents were sent letters explaining the purpose of the study, and that students in selected math classes (Algebra I and II, Geometry) were eligible to participate in the study. In study 1, parents were instructed that they could opt out, and their students would not participate. Students enrolled in the study were eligible for compensation via gift card raffles for completing follow-up surveys associated with the study. In study 2, parents provided informed written consent for their student to participate in the study, and student participants were not permitted to be compensated for participating in the study. Across both studies, before the start of the intervention, all students read an assent statement and provided verbal assent to participate in study procedures.
Ethics oversight	Local administration at each high school (Superintendent, local principal, school district IRB) approved the study procedures, as well as the IRB of record, the Committee for the Protection of Human Subjects at Dartmouth College (#28333).

Note that full information on the approval of the study protocol must also be provided in the manuscript.

Field-specific reporting

Please select the one below that is the best fit for your research. If you are not sure, read the appropriate sections before making your selection.

☐ Life sciences ☐ Behavioural & social sciences ☐ Ecological, evolutionary & environmental sciences

For a reference copy of the document with all sections, see nature.com/documents/nr-reporting-summary-flat.pdf

Life sciences study design

All studies must disclose on these points even when the disclosure is negative.

Sample size	*Describe how sample size was determined, detailing any statistical methods used to predetermine sample size OR if no sample-size calculation was performed, describe how sample sizes were chosen and provide a rationale for why these sample sizes are sufficient.*
Data exclusions	*Describe any data exclusions. If no data were excluded from the analyses, state so OR if data were excluded, describe the exclusions and the rationale behind them, indicating whether exclusion criteria were pre-established.*
Replication	*Describe the measures taken to verify the reproducibility of the experimental findings. If all attempts at replication were successful, confirm this OR if there are any findings that were not replicated or cannot be reproduced, note this and describe why.*

Randomization	*Describe how samples/organisms/participants were allocated into experimental groups. If allocation was not random, describe how covariates were controlled OR if this is not relevant to your study, explain why.*
Blinding	*Describe whether the investigators were blinded to group allocation during data collection and/or analysis. If blinding was not possible, describe why OR explain why blinding was not relevant to your study.*

Behavioural & social sciences study design

All studies must disclose on these points even when the disclosure is negative.

Study description	This study includes a longitudinal quantitative design, with measures of grade performance and survey responses collected at multiple points throughout a school year. Additional analyses of some qualitative data that was collected incidentally as a part of the activities given to the students during the study is included in the Supplementary Material for this study.
Research sample	Participants in this study were recruited from two different school districts in geographically distinct regions of the US. School 1 was a small high school in rural New England. Classes were taught along different timescales: including semester-long, and year-long courses. At School 1, all students enrolled in these classes were invited to participate and parents were asked to opt-out of the study if they chose not to participate (parents were sent a letter informing of the study prior to the start of the study). The local superintendent, school administration, and Dartmouth Committee for the Protection of Human Subjects (CPHS #28333) approved these procedures that a written informed consent was not needed and that an opt-out sampling protocol was approved. No parents opted-out of the study. All students provided verbal assent to complete study procedures. One-hundred-nine adolescent participants were recruited for the study from their math classes (algebra I and II, geometry) taught by two instructors. From this sample, two students opted-out of the surveys, and 16 students had incomplete survey or grade data due to absences or technical difficulties with the online surveys. From the overall sample of N = 91 across six classes, students were between the ages of 13 and 18 (Mage = 15.34, SDage = 1.05), and the sample was 60% female. Demographic information was provided by each school. For our analyses, we use the term “gender” to refer to masculine or feminine identity, as this encompasses the cultural and social context of gender roles, and our study does not refer to any biological measures of sex characteristics. The researchers realize there are a variety of identities that can be encompassed by gender identity, but for the purposes of these analyses, the researchers use the binary terms “male” and “female” to refer to the participants’ gender identity as this was the information provided by each school. For additional information about this sample, please see the Supplementary Material.<br>At School 2, approximately 272 students from a diverse high school in the mid-Atlantic region were invited to participate in this study, and parents provided a signed consent form for their student to opt-in to enroll in the study. Students were enrolled in Algebra I, Algebra II, and Algebra II honors classes, and were recruited from 13 year-long classes taught by six instructors. Out of this sample, 167 students had parents who provided a signed informed consent in order to participate in the study (59% response rate, 6 students not enrolled because they were enrolled in a different math class). In addition to the consent provided by parents, all students gave verbal assent. A total of 156 students were included in the dataset for analysis after an additional 5 students were excluded for incomplete grade information (N = 156, 53% female, Mage = 15.46, SDage = .92, Rangeage = 14-19; n = 21 Algebra I students, 43% female; n = 100 Algebra II students, 58% female; n = 34 Algebra II honors students, 44% female). For more information about the demographics of this sample, please see the Supplementary Material.
Sampling strategy	For both schools, we chose to recruit adolescent students enrolled in Algebra I and II (and geometry at school 1). These students were all enrolled in math courses that were required to graduate from high school. All students enrolled in these courses were invited to participate. We did not conduct an a priori power analysis to compute suggested sample size for this study. However, our sample was judged to be sufficient, given the within-subject nature of our comparisons, and our sampling from two diverse student bodies from separate regions of the country was thought to provide a sufficient number of students with sufficient diversity to ensure our results could be interpretable.
Data collection	At school 1, student data was collected via a combination of online and pencil-and-paper forms completed by the participant. Participants completed a variety of different self-report scales, as well as short follow-up assessments. Participants completed a worksheet during a semi-structured small-group discussion for their in-class intervention. In addition, students completed paper reminders for follow-up throughout the rest of second semester. At school 2, students completed the same assessments, but all were completed via paper and pencil worksheets. For both schools, the school provided demographic data, and information about school performance including grades.
Timing	For School 1, data collection was conducted during the 2015-2016 school year. For school 2, data collection was conducted during the 2016-2017 school year. For both schools data was provided by the school for the first semester, and the intervention was introduced during the second semester.
Data exclusions	For school 1, one-hundred-nine adolescent participants were recruited for the study from their math classes (algebra I and II, geometry) taught by two instructors. From this sample, two students opted-out of the surveys, and 16 students had incomplete survey or grade data due to absences or technical difficulties with the online surveys. From the overall sample of N = 91 across six classes, students were between the ages of 13 and 18 (Mage = 15.34, SDage = 1.05), and the sample was 60% female. For school 2, At School 2, approximately 272 students from a diverse high school in the mid-Atlantic region were invited to participate in this study, and parents provided a signed consent form for their student to opt-in to enroll in the study. Students were enrolled in Algebra I, Algebra II, and Algebra II honors classes, and were recruited from 13 year-long classes taught by six instructors. Out of this sample, 167 students had parents who provided a signed informed consent in order to participate in the study (59% response rate, 6 students not enrolled because they were enrolled in a different math class). In addition to the consent provided by parents, all students gave verbal assent. A total of 156 students were included in the dataset for analysis after an additional 5 students were excluded for incomplete grade information (N = 156, 53% female, Mage = 15.46, SDage = .92, Rangeage = 14-19; n = 21 Algebra I students, 43% female; n = 100 Algebra II students, 58% female; n = 34 Algebra II honors students, 44% female).
Non-participation	At school 1, the school approved an opt-out procedure for consent/assent. Two students decided to opt-out of the study and

Non-participation	declined participation. At school 2, which required written consent from parents in order to participate. From the pool of eligible students, N=167 provided written informed consent (59% response rate). No students declined participation in the study in this sample.
Randomization	At both schools, students were pseudo-randomly assigned to the intervention group. Classes were split in half, with half of the eligible students assigned to one intervention strategy, and the other half assigned to the other. Groups were shuffled so that the distribution of male and female students was roughly counterbalanced between each intervention group in the class. Assignment to intervention groups were counterbalanced for gender, previous grade performance (School 2) and/or GPA and/or standardized test performance (School 1). Half of the students were assigned to an intervention technique focused on improving study skills (SS), half were assigned to an intervention technique focused on emotion regulation (ER) using cognitive reappraisal. In this way, we were able to establish that even within each class, the intervention groups would be relatively balanced in terms of gender composition and academic performance before the intervention was introduced.

Ecological, evolutionary & environmental sciences study design

All studies must disclose on these points even when the disclosure is negative.

Study description	*Briefly describe the study. For quantitative data include treatment factors and interactions, design structure (e.g. factorial, nested, hierarchical), nature and number of experimental units and replicates.*
Research sample	*Describe the research sample (e.g. a group of tagged Passer domesticus, all Stenocereus thurberi within Organ Pipe Cactus National Monument), and provide a rationale for the sample choice. When relevant, describe the organism taxa, source, sex, age range and any manipulations. State what population the sample is meant to represent when applicable. For studies involving existing datasets, describe the data and its source.*
Sampling strategy	*Note the sampling procedure. Describe the statistical methods that were used to predetermine sample size OR if no sample-size calculation was performed, describe how sample sizes were chosen and provide a rationale for why these sample sizes are sufficient.*
Data collection	*Describe the data collection procedure, including who recorded the data and how.*
Timing and spatial scale	*Indicate the start and stop dates of data collection, noting the frequency and periodicity of sampling and providing a rationale for these choices. If there is a gap between collection periods, state the dates for each sample cohort. Specify the spatial scale from which the data are taken*
Data exclusions	*If no data were excluded from the analyses, state so OR if data were excluded, describe the exclusions and the rationale behind them, indicating whether exclusion criteria were pre-established.*
Reproducibility	*Describe the measures taken to verify the reproducibility of experimental findings. For each experiment, note whether any attempts to repeat the experiment failed OR state that all attempts to repeat the experiment were successful.*
Randomization	*Describe how samples/organisms/participants were allocated into groups. If allocation was not random, describe how covariates were controlled. If this is not relevant to your study, explain why.*
Blinding	*Describe the extent of blinding used during data acquisition and analysis. If blinding was not possible, describe why OR explain why blinding was not relevant to your study.*

Did the study involve field work? ☐ Yes ☐ No

Field work, collection and transport

Field conditions	*Describe the study conditions for field work, providing relevant parameters (e.g. temperature, rainfall).*
Location	*State the location of the sampling or experiment, providing relevant parameters (e.g. latitude and longitude, elevation, water depth).*
Access & import/export	*Describe the efforts you have made to access habitats and to collect and import/export your samples in a responsible manner and in compliance with local, national and international laws, noting any permits that were obtained (give the name of the issuing authority, the date of issue, and any identifying information).*
Disturbance	*Describe any disturbance caused by the study and how it was minimized.*

Reporting for specific materials, systems and methods

We require information from authors about some types of materials, experimental systems and methods used in many studies. Here, indicate whether each material, system or method listed is relevant to your study. If you are not sure if a list item applies to your research, read the appropriate section before selecting a response.

Materials & experimental systems

n/a	Involved in the study
☐	☐ Antibodies
☐	☐ Eukaryotic cell lines
☐	☐ Palaeontology and archaeology
☐	☐ Animals and other organisms
☐	☐ Clinical data
☐	☐ Dual use research of concern
☐	☐ Plants

Methods

n/a	Involved in the study
☐	☐ ChIP-seq
☐	☐ Flow cytometry
☐	☐ MRI-based neuroimaging

Antibodies

Antibodies used

Describe all antibodies used in the study; as applicable, provide supplier name, catalog number, clone name, and lot number.

Validation

Describe the validation of each primary antibody for the species and application, noting any validation statements on the manufacturer's website, relevant citations, antibody profiles in online databases, or data provided in the manuscript.

Eukaryotic cell lines

Policy information about cell lines and Sex and Gender in Research

Cell line source(s)

State the source of each cell line used and the sex of all primary cell lines and cells derived from human participants or vertebrate models.

Authentication

Describe the authentication procedures for each cell line used OR declare that none of the cell lines used were authenticated.

Mycoplasma contamination

Confirm that all cell lines tested negative for mycoplasma contamination OR describe the results of the testing for mycoplasma contamination OR declare that the cell lines were not tested for mycoplasma contamination.

Commonly misidentified lines
(See ICLAC register)

Name any commonly misidentified cell lines used in the study and provide a rationale for their use.

Palaeontology and Archaeology

Specimen provenance

Provide provenance information for specimens and describe permits that were obtained for the work (including the name of the issuing authority, the date of issue, and any identifying information). Permits should encompass collection and, where applicable, export.

Specimen deposition

Indicate where the specimens have been deposited to permit free access by other researchers.

Dating methods

If new dates are provided, describe how they were obtained (e.g. collection, storage, sample pretreatment and measurement), where they were obtained (i.e. lab name), the calibration program and the protocol for quality assurance OR state that no new dates are provided.

☐ Tick this box to confirm that the raw and calibrated dates are available in the paper or in Supplementary Information.

Ethics oversight

Identify the organization(s) that approved or provided guidance on the study protocol, OR state that no ethical approval or guidance was required and explain why not.

Note that full information on the approval of the study protocol must also be provided in the manuscript.

Animals and other research organisms

Policy information about studies involving animals; ARRIVE guidelines recommended for reporting animal research, and Sex and Gender in Research

Laboratory animals

For laboratory animals, report species, strain and age OR state that the study did not involve laboratory animals.

Wild animals

Provide details on animals observed in or captured in the field; report species and age where possible. Describe how animals were caught and transported and what happened to captive animals after the study (if killed, explain why and describe method; if released, say where and when) OR state that the study did not involve wild animals.

Reporting on sex

Indicate if findings apply to only one sex; describe whether sex was considered in study design, methods used for assigning sex. Provide data disaggregated for sex where this information has been collected in the source data as appropriate; provide overall

	numbers in this Reporting Summary. Please state if this information has not been collected. Report sex-based analyses where performed, justify reasons for lack of sex-based analysis.
Field-collected samples	*For laboratory work with field-collected samples, describe all relevant parameters such as housing, maintenance, temperature, photoperiod and end-of-experiment protocol OR state that the study did not involve samples collected from the field.*
Ethics oversight	*Identify the organization(s) that approved or provided guidance on the study protocol, OR state that no ethical approval or guidance was required and explain why not.*

Note that full information on the approval of the study protocol must also be provided in the manuscript.

Clinical data

Policy information about clinical studies

All manuscripts should comply with the ICMJE guidelines for publication of clinical research and a completed CONSORT checklist must be included with all submissions.

Clinical trial registration	*Provide the trial registration number from ClinicalTrials.gov or an equivalent agency.*
Study protocol	*Note where the full trial protocol can be accessed OR if not available, explain why.*
Data collection	*Describe the settings and locales of data collection, noting the time periods of recruitment and data collection.*
Outcomes	*Describe how you pre-defined primary and secondary outcome measures and how you assessed these measures.*

Dual use research of concern

Policy information about dual use research of concern

Hazards

Could the accidental, deliberate or reckless misuse of agents or technologies generated in the work, or the application of information presented in the manuscript, pose a threat to:

No	Yes	
☐	☐	Public health
☐	☐	National security
☐	☐	Crops and/or livestock
☐	☐	Ecosystems
☐	☐	Any other significant area

Experiments of concern

Does the work involve any of these experiments of concern:

No	Yes	
☐	☐	Demonstrate how to render a vaccine ineffective
☐	☐	Confer resistance to therapeutically useful antibiotics or antiviral agents
☐	☐	Enhance the virulence of a pathogen or render a nonpathogen virulent
☐	☐	Increase transmissibility of a pathogen
☐	☐	Alter the host range of a pathogen
☐	☐	Enable evasion of diagnostic/detection modalities
☐	☐	Enable the weaponization of a biological agent or toxin
☐	☐	Any other potentially harmful combination of experiments and agents

Plants

Seed stocks	*Report on the source of all seed stocks or other plant material used. If applicable, state the seed stock centre and catalogue number. If plant specimens were collected from the field, describe the collection location, date and sampling procedures.*
Novel plant genotypes	*Describe the methods by which all novel plant genotypes were produced. This includes those generated by transgenic approaches, gene editing, chemical/radiation-based mutagenesis and hybridization. For transgenic lines, describe the transformation method, the number of independent lines analyzed and the generation upon which experiments were performed. For gene-edited lines, describe the editor used, the endogenous sequence targeted for editing, the targeting guide RNA sequence (if applicable) and how the editor*

was applied.

Authentication

Describe any authentication procedures for each seed stock used or novel genotype generated. Describe any experiments used to assess the effect of a mutation and, where applicable, how potential secondary effects (e.g. second site T-DNA insertions, mosiacism, off-target gene editing) were examined.

ChIP-seq

Data deposition

- [ ] Confirm that both raw and final processed data have been deposited in a public database such as GEO.
- [ ] Confirm that you have deposited or provided access to graph files (e.g. BED files) for the called peaks.

Data access links<br>*May remain private before publication.*	*For "Initial submission" or "Revised version" documents, provide reviewer access links. For your "Final submission" document, provide a link to the deposited data.*
Files in database submission	*Provide a list of all files available in the database submission.*
Genome browser session<br>(e.g. UCSC)	*Provide a link to an anonymized genome browser session for "Initial submission" and "Revised version" documents only, to enable peer review. Write "no longer applicable" for "Final submission" documents.*

Methodology

Replicates	*Describe the experimental replicates, specifying number, type and replicate agreement.*
Sequencing depth	*Describe the sequencing depth for each experiment, providing the total number of reads, uniquely mapped reads, length of reads and whether they were paired- or single-end.*
Antibodies	*Describe the antibodies used for the ChIP-seq experiments; as applicable, provide supplier name, catalog number, clone name, and lot number.*
Peak calling parameters	*Specify the command line program and parameters used for read mapping and peak calling, including the ChIP, control and index files used.*
Data quality	*Describe the methods used to ensure data quality in full detail, including how many peaks are at FDR 5% and above 5-fold enrichment.*
Software	*Describe the software used to collect and analyze the ChIP-seq data. For custom code that has been deposited into a community repository, provide accession details.*

Flow Cytometry

Plots

Confirm that:

- [ ] The axis labels state the marker and fluorochrome used (e.g. CD4-FITC).
- [ ] The axis scales are clearly visible. Include numbers along axes only for bottom left plot of group (a 'group' is an analysis of identical markers).
- [ ] All plots are contour plots with outliers or pseudocolor plots.
- [ ] A numerical value for number of cells or percentage (with statistics) is provided.

Methodology

Sample preparation	*Describe the sample preparation, detailing the biological source of the cells and any tissue processing steps used.*
Instrument	*Identify the instrument used for data collection, specifying make and model number.*
Software	*Describe the software used to collect and analyze the flow cytometry data. For custom code that has been deposited into a community repository, provide accession details.*
Cell population abundance	*Describe the abundance of the relevant cell populations within post-sort fractions, providing details on the purity of the samples and how it was determined.*
Gating strategy	*Describe the gating strategy used for all relevant experiments, specifying the preliminary FSC/SSC gates of the starting cell population, indicating where boundaries between "positive" and "negative" staining cell populations are defined.*

- [ ] Tick this box to confirm that a figure exemplifying the gating strategy is provided in the Supplementary Information.

Magnetic resonance imaging

Experimental design

Design type	*Indicate task or resting state; event-related or block design.*
Design specifications	*Specify the number of blocks, trials or experimental units per session and/or subject, and specify the length of each trial or block (if trials are blocked) and interval between trials.*
Behavioral performance measures	*State number and/or type of variables recorded (e.g. correct button press, response time) and what statistics were used to establish that the subjects were performing the task as expected (e.g. mean, range, and/or standard deviation across subjects).*

Acquisition

Imaging type(s)	*Specify: functional, structural, diffusion, perfusion.*
Field strength	*Specify in Tesla*
Sequence & imaging parameters	*Specify the pulse sequence type (gradient echo, spin echo, etc.), imaging type (EPI, spiral, etc.), field of view, matrix size, slice thickness, orientation and TE/TR/flip angle.*
Area of acquisition	*State whether a whole brain scan was used OR define the area of acquisition, describing how the region was determined.*

Diffusion MRI ☐ Used ☐ Not used

Preprocessing

Preprocessing software	*Provide detail on software version and revision number and on specific parameters (model/functions, brain extraction, segmentation, smoothing kernel size, etc.).*
Normalization	*If data were normalized/standardized, describe the approach(es): specify linear or non-linear and define image types used for transformation OR indicate that data were not normalized and explain rationale for lack of normalization.*
Normalization template	*Describe the template used for normalization/transformation, specifying subject space or group standardized space (e.g. original Talairach, MNI305, ICBM152) OR indicate that the data were not normalized.*
Noise and artifact removal	*Describe your procedure(s) for artifact and structured noise removal, specifying motion parameters, tissue signals and physiological signals (heart rate, respiration).*
Volume censoring	*Define your software and/or method and criteria for volume censoring, and state the extent of such censoring.*

Statistical modeling & inference

Model type and settings	*Specify type (mass univariate, multivariate, RSA, predictive, etc.) and describe essential details of the model at the first and second levels (e.g. fixed, random or mixed effects; drift or auto-correlation).*
Effect(s) tested	*Define precise effect in terms of the task or stimulus conditions instead of psychological concepts and indicate whether ANOVA or factorial designs were used.*

Specify type of analysis: ☐ Whole brain ☐ ROI-based ☐ Both

Statistic type for inference (See Eklund et al. 2016)	*Specify voxel-wise or cluster-wise and report all relevant parameters for cluster-wise methods.*
Correction	*Describe the type of correction and how it is obtained for multiple comparisons (e.g. FWE, FDR, permutation or Monte Carlo).*

Models & analysis

n/a	Involved in the study
☐	☐ Functional and/or effective connectivity
☐	☐ Graph analysis
☐	☐ Multivariate modeling or predictive analysis

Functional and/or effective connectivity	*Report the measures of dependence used and the model details (e.g. Pearson correlation, partial correlation, mutual information).*
Graph analysis	*Report the dependent variable and connectivity measure, specifying weighted graph or binarized graph,*

Graph analysis | *subject- or group-level, and the global and/or node summaries used (e.g. clustering coefficient, efficiency, etc.).*

Multivariate modeling and predictive analysis | *Specify independent variables, features extraction and dimension reduction, model, training and evaluation metrics.*
